# PPM1K mediates metabolic disorder of branched-chain amino acid and regulates cerebral ischemia-reperfusion injury by activating ferroptosis in neurons

**DOI:** 10.1038/s41419-023-06135-x

**Published:** 2023-09-26

**Authors:** Tao Li, Lili Zhao, Ye Li, Meijuan Dang, Jialiang Lu, Ziwei Lu, Qiao Huang, Yang Yang, Yuxuan Feng, Xiaoya Wang, Yating Jian, Heying Wang, Yingying Guo, Lei Zhang, Yu Jiang, Songhua Fan, Shengxi Wu, Hong Fan, Fang Kuang, Guilian Zhang

**Affiliations:** 1 Department of Neurology, the Second Affiliated Hospital of Xi’an Jiaotong University, Xi’an, 710004 Shaanxi China; 2 Department of Neurobiology, School of Basic Medicine, Fourth Military Medical University, Xi’an, 710032 Shaanxi China; 3 Department of Pediatrics, the Second Affiliated Hospital of Xi’an Jiaotong University, Xi’an, 710004 Shaanxi China

**Keywords:** Stroke, Cell death in the nervous system

## Abstract

Ischemic stroke is a neurological disorder caused by vascular stenosis or occlusion, accounting for approximately 87% of strokes. Clinically, the most effective therapy for ischemic stroke is vascular recanalization, which aims to rescue neurons undergoing ischemic insults. Although reperfusion therapy is the most effective treatment for ischemic stroke, it still has limited benefits for many patients, and ischemia-reperfusion (I/R) injury is a widely recognized cause of poor prognosis. Here, we aim to investigate the mechanism of protein phosphatase Mg^2+^/Mn^2+^ dependent 1 K (PPM1K) mediates metabolic disorder of branched-chain amino acids (BCAA) by promoting fatty acid oxidation led to ferroptosis after cerebral I/R injury. We established the I/R model in mice and used BT2, a highly specific BCAA dehydrogenase (BCKD) kinase inhibitor to promote BCAA metabolism. It was further verified by lentivirus knocking down PPM1K in neurons. We found that BCAA levels were elevated after I/R injury due to dysfunctional oxidative degradation caused by phosphorylated BCKD E1α subunit (BCKDHA). Additionally, the level of phosphorylated BCKDHA was determined by decreased PPM1K in neurons. We next demonstrated that BCAA could induce oxidative stress, lipid peroxidation, and ferroptosis in primary cultured cortical neurons in vitro. Our results further showed that BT2 could reduce neuronal ferroptosis by enhancing BCAA oxidation through inhibition of BCKDHA phosphorylation. We further found that defective BCAA catabolism could induce neuronal ferroptosis by PPM1K knockdown. Furthermore, BT2 was found to alleviate neurological behavior disorders after I/R injury in mice, and the effect was similar to ferroptosis inhibitor ferrostatin-1. Our findings reveal a novel role of BCAA in neuronal ferroptosis after cerebral ischemia and provide a new potential target for the treatment of ischemic stroke.

## Introduction

Stroke is a significant cause of death and disability in the world, with approximately 795,000 people suffering from new or recurrent stroke every year, approximately 87% of which are ischemic strokes [1]. Reperfusion therapy (including intravenous thrombolysis and arterial thrombectomy) represents a milestone in the treatment of ischemic stroke and is currently the most effective treatment. It aims to rescue injured neurons by restoring blood flow. Nevertheless, a good prognosis does not match the reestablishment of cerebral blood flow. Ischemia-reperfusion (I/R) injury, which triggers a rapid cascade of neuropathological events including cellular bioenergetics and redox homeostasis, is a widely recognized cause of this disparity [2]. I/R involves reoxygenation-induced production of reactive oxygen species (ROS), calcium overload, inflammatory responses, endoplasmic reticulum stress, and cell death [3]. However, the underlying mechanisms of I/R injury are not yet fully understood.

Neuronal death is significant in I/R injury and the main cause of neurological deficits. Thus, uncovering the mechanism of neuronal injury and loss is urgently needed. Ferroptosis, a novel form of regulated cell death, is characterized by the accumulation of iron-mediated lipid peroxide of polyunsaturated fatty acids (PUFA) [4]. The biological characteristics of ferroptosis include metabolic dysfunction of iron ions, depletion of glutathione (GSH), and accumulation of lipid ROS [4, 5]. Ferroptosis is one of the main forms of neuronal cell death in central nervous system injuries and neurodegenerative diseases [4, 6]. Ferrostatin-1 (Fer-1) and liproxstatin-1 can reportedly mitigate I/R-induced damage in the brain by inhibiting ferroptosis [7, 8].

Metabolic dysfunction of amino acids is closely related to the occurrence of ferroptosis [9] and tightly linked to cerebral I/R injury. Branched-chain amino acids (BCAA), including leucine, isoleucine, and valine, are indispensable nutrients for human beings. They share similar structural features, such as short aliphatic side chains, and have a common catabolic pathway; indeed, the first two steps in their catabolism are catalyzed by the same enzymes [10]. High levels of BCAA or branched-chain alpha-keto acids (BCKA) reduced total antioxidant reactivity and significantly increased the production of nitric oxide and lipid peroxidation products, eliciting cell death in vitro and in vivo [11–14]. Our previous metabolomics study [15] indicated markedly elevated of BCAA levels after cerebral ischemia. However, whether BCAA is involved in neuronal ferroptosis after cerebral I/R injury remains unclear. Inspired by these findings, we investigated the role and underlying mechanism of BCAA in ferroptosis after cerebral I/R in this study. Our findings demonstrate that BCAA mediated neuronal ferroptosis in cerebral I/R injury, providing novel insight for understanding I/R injury and treatment of ischemic stroke.

## Materials and methods

### Animals and experimental protocol

Adult male C57BL/6 mice (20–25 g, 6–8 weeks) and Sprague-Dawley rats (250–300 g, 6–8 weeks) were housed in a pathogen-free facility with a 12-h light/dark cycle and allowed free access to food and water. Rats were randomly assigned to the middle cerebral artery occlusion (MCAO) group or sham group and underwent metabolomics analysis after surgery.

We used 3,6-dichlorobenzo[b]thiophene-2-carboxylic acid (BT2) or Fer-1 to assess the influence of BCAA and ferroptosis on cerebral I/R damage in mice. BT2 (25643, Chem-Impex, Wood Dale, IL, USA) and Fer-1 (SML0583, Sigma-Aldrich, St. Louis, MO, USA) were dissolved in a vehicle buffer (VEH) containing 5% dimethyl sulfoxide (DMSO, D2650, Sigma-Aldrich), 10% cremophor EL (HY-Y1890, MedChemExpress, Monmouth Junction, NJ, USA), and 85% sodium bicarbonate (0.1 M, pH 9.0). BT2 was injected intraperitoneally (i.p.) at a dosage of 40 mg/kg/day, as previously described [16]. Fer-1 was administered i.p. at a dosage of 10 mg/kg/day [17]. Mice were injected with BT2, Fer-1, or VEH twice (at 24 h and 2 h) before cerebral I/R. Mice were randomly divided into five groups: (1) sham group mice underwent surgery without MCAO/R, (2) I/R group mice received MCAO, (3) VEH group mice underwent MCAO/R and received VEH, (4) BT2 group mice underwent MCAO/R and received BT2, and (5) Fer-1 group mice underwent MCAO/R and received Fer-1. All animal experiments were approved by the Committee on the Ethics of Animal Experiments of the College of Medicine, Xi’an Jiaotong University and performed in line with the principles of the Declaration of Helsinki.

### MCAO model

MCAO and cerebral I/R models were established as previously described [7, 18]. Rats or mice were anesthetized with isoflurane (5% induction and 1.5% maintenance). To avoid death due to hypothermia during and after the operation, the temperature of animals was maintained at 37 °C. Right unilateral MCAO was accomplished by inserting a silicon rubber-coated nylon monofilament (3600, Guangzhou Jialing Biotechnology, Guangzhou, China) into the internal carotid artery (ICA) via the common carotid artery, and advancing the filament 20–21 mm (in rats) past the carotid bifurcation until a slight resistance was felt. Similarly, a 3-0 nylon monofilament wire (MSMC21B120PK50, RWD Life Science, Shenzhen, China) was inserted from the right external carotid artery into the right ICA and advanced 9–10 mm in mice. For cerebral I/R modeling, the occluding filament was subsequently withdrawn for reperfusion after arterial occlusion for 60 min in mice. Sham-operated mice or rats only had their blood vessels isolated without inserting a nylon wire.

After surgery, animals were initially examined for neurological deficits when fully awake according to Longa’s five-tiered grading system, as follows: 0, no deficit; 1, failure to extend contralateral forepaw; 2, spin longitudinally; 3, falling to the right side; 4, unable to walk spontaneously. A score of 1–3 points was the criterion for successful modeling [19].

### Experimental sample preparation

After anesthetizing mice at the indicated time points with 1% pentobarbital sodium (100 mg/kg. i.p.), samples were quickly collected. For tissue sampling, the ischemic border tissue was rapidly separated. The ischemic border was defined as the approximately 500-μm-wide region containing generally morphologically normal cells that surrounds the core [20]. Tissues and serum samples were placed in Eppendorf tubes and stored at −80 °C. To observe morphological changes, mice were first perfused with 20 mL of phosphate-buffered saline (PBS, 0.01 M), followed by 40 mL of 4% paraformaldehyde (PFA). Next, brains were removed and postfixed in 4% PFA, followed by dehydration in 30% sucrose. After embedding in OCT compound (4583, Sakura, CA, USA), brains were sliced (Leica CM1950 Cryostat, Wetzlar, Germany) into 20-μm-thick coronal sections.

### LC-MS and GC-MS analysis of metabolomics

Rat brain samples were harvested 24 h after operation in each of the MCAO and sham groups (six rats per group). Liquid chromatography-mass spectrometry (LC-MS) and gas chromatography-mass spectrometry (GC-MS) were performed as we previously described [15]. The ultra-high performance liquid chromatography (UHPLC) separation was carried out using a 1290 Infinity series UHPLC System (Agilent Technologies). The TripleTOF 6600 mass spectrometry (AB Sciex) was used for its ability to acquire MS/MS spectra on an information-dependent basis (IDA) during an LC-MS experiment. In each cycle, the most intensive 12 precursor ions with intensity above 100 were chosen for MS/MS at collision energy (CE) of 30 eV. The cycle time was 0.56 s. ESI source conditions were set as following: Gas 1 as 60 psi, Gas 2 as 60 psi, Curtain Gas as 35 psi, Source Temperature as 600 °C, Declustering potential as 60 V, Ion Spray Voltage Floating (ISVF) as 5000 V or −4000 V in positive or negative modes, respectively. GC-TOF-MS analysis was performed using an Agilent 7890 gas chromatograph coupled with a time-of-flight mass spectrometer. The system utilized a DB-5MS capillary column. 1 μL aliquot of sample was injected in splitless mode. Helium was used as the carrier gas, the front inlet purge flow was 3 mL min^−1^, and the gas flow rate through the column was 1 mL min^−1^. The initial temperature was kept at 50 °C for 1 min, then raised to 310 °C at a rate of 10 °C min^−1^, then kept for 8 min at 310 °C. The injection, transfer line, and ion source temperatures were280, 280 and 250 °C, respectively. The energy was −70 eV in electron impact mode. The mass spectrometry data were acquired in full-scan mode with the m/z range of 50–500 at a rate of 12.5 spectra per second after a solvent delay of 6.30 min [21].

### Measurement of BCAA concentration

After reperfusion, brain tissues and serum were immediately collected for detection of BCAA or stored at −80 °C. Tissues were homogenized according to the manufacturer’s instructions, and the supernatant was analyzed with a commercial kit (K564-100, Biovision, Milpitas, CA, USA) to determine relative levels of BCAA. The absorbance of each sample was measured at 450 nm with a multimode microplate reader (Infinite M200, Tecan, Männedorf, Switzerland) and calculated based on the standard curve.

### Real-time quantitative polymerase chain reaction (qRT-PCR)

Total RNA was extracted and purified from cultured cells or tissues using TRizol (15596018, Life Technologies, Waltham, MA, USA). A reverse transcriptase kit (RR036A-1, Takara, Kusatsu, Japan) was used to reverse total RNA into cDNA. qRT-PCR was performed on a Bio-Rad CFX system (Hercules, CA, USA) using SYBR Green Master Mix (RR820A, Takara). All gene expression values were normalized to β-actin mRNA levels in each sample using the 2^−∆∆Ct^ method. Primer sequences are listed in Supplementary Table S1.

### Western blotting

Total protein was extracted and purified from brain tissues or cultured cells with radioimmunoprecipitation assay lysis buffer according to the manufacturer’s instructions. Proteins were separated in sodium dodecyl sulfate polyacrylamide gels and transferred onto a polyvinylidene difluoride membrane, which was blocked with 5% nonfat milk. The following primary antibodies were used overnight for blots: BCAA dehydrogenase (BCKD; AF0314, 1:500, Affinity Biosciences, Cincinnati, OH, USA), BCKD kinase (BCKDK; sc-374435, 1:500, Santa Cruz Biotechnology, Dallas, TX, USA), BCKD E1α subunit (BCKDHA) phosphorylated S293 (p-BCKDHA S293; ab200577, 1:5000, Abcam, Cambridge, UK), BCKDHA (90198, 1:1000, Cell Signaling Technology), protein phosphatase Mg2^+^/Mn2^+^ dependent 1 K (PPM1K; ab135286, 1:1000, Abcam), Acyl-CoA synthetase long chain family member 4 (ACSL4; ab155282, 1:5000, Abcam), prostaglandin-endoperoxide synthase 2 (PTGS2; 12375-1-AP, 1:1000, Proteintech, Rosemont, IL, USA), xCT (ab175186, 1:1000, Abcam), ferritin heavy chain (FTH1; 3998, 1:1000, Cell Signaling Technology), glutathione peroxidase 4 (GPX4; ab125066, 1:1000, Abcam), Ferroportin (FPN, 26601-1-AP, 1:500, Proteintech), Transferrin receptor (TFRC, NB100-92243, 1:500, Novus Biologicals, Centennial, CO, USA), and β-actin (A5441, 1:10000, Sigma-Aldrich) at 4 °C. After incubation with appropriate horseradish peroxidase-conjugated secondary antibodies (A21202, A21203, A21206, A21207, A21209, 1:500, Life Technologies) for 2 h, bands were developed and visualized using VisionWorks (Analytik Jena, Jena, Germany). The results were analyzed with ImageJ software (National Institutes of Health, Bethesda, MD, USA).

### Immunohistochemistry

After washing with PBS, sections were blocked with 10% donkey serum, 0.5% bovine serum albumin (BSA), and 0.25% Triton X-100 in PBS for 1 h. Next, sections were incubated with the following primary antibodies overnight at 4 °C: PPM1K (1:200), p-BCKDHA S293 (1:200), 4-hydroxynonena (4-HNE; MHN-100P, 1:50, Japan Institute for the Control of Aging, Fukuroi, Japan), ionized calcium binding adapter molecule 1 (Iba1; 019-19741, 1:1000, Wako Chemicals, Richmond, VA, USA), glial fibrillary acidic protein (GFAP, Z0334, 1:1000, Dako, Glostrup, Denmark), and neuronal nuclei (NeuN; MAB377, 1:1000, Sigma-Aldrich; ABN78, 1:1000, Sigma-Aldrich; OB-PRT013, 1:500, Oasisbiofarm, Las Vegas, NV, USA). Subsequently, slides were incubated with corresponding Alexa Fluor 594-, Alexa Fluor 488-, or Alexa Fluor 647-labeled secondary antibodies. After counterstaining with 4’,6-diamidino-2-phenylindole (DAPI, D9564, Sigma-Aldrich) for 10 min, slides were washed three times with PBS and covered using 50% glycerol in PBS. Images were subsequently taken using a confocal microscope (FV 3000, Olympus, Tokyo, Japan) and quantitatively analyzed by ImageJ software. Sections from four animals in each group were immunohistochemically stained and used for double- or triple-labeling experiments.

### Measurement of MDA, GSH, and Iron

To quantify levels of malondialdehyde (MDA), GSH, and iron, tissues, and cells were collected and assayed with kits according to the manufacturer’s instructions. Samples were homogenized after reperfusion or culture. After centrifuging at 4 °C and 1.2 × 10^4 ^g for 10 min, the supernatant of samples was used to detect contents of MDA (S0131; Beyotime Biotechnology, Haimen, China), GSH, oxidized GSH (GSSG, S0053, Beyotime), and iron levels (A039-2-1; Nanjing Jiancheng Bioengineering Institute, Nanjing, China).

### Primary cortical neuron culture

Primary cortical neurons were isolated from embryonic day 15 (E15)–E16 mouse embryos as previously described [22]. In brief, the cortex was dissected and digested in 0.25% trypsin for 10 min at 37 °C. Neurons were seeded on the poly-L-lysine coated plates or dishes in Neurobasal medium (21103-049, Gibco) supplemented with 1% B27(17504-044, Gibco), 1% Glutamine (25030-081, Gibco) and 1% penicillin-streptomycin (15140-122, Gibco) for 6–7 days. After that, we changed the culture medium half every 3 days.

### Cell treatment

Neurons were cultured for 24 h with BCAA (leucine:valine:isoleucine = 2:1:1; L8912, I7403, V1255, Sigma-Aldrich) at 2.5, 5, 10, 20, 50, and 100 mmol/L (mM) in the medium. RSL3 (5 mM; S8155; Selleck Chemicals, Houston, TX, USA) and erastin (10 mM; S7242, Selleck Chemicals) stock solutions were dissolved in DMSO at a storage concentration. These reagents were added into the medium for 2 h at 5 μM (RSL3) or 10 μM (erastin) to induce ferroptosis in vitro. Cells were pretreated with BT2 (80 μM, dissolved in DMSO) or Fer-1 (5 μM, dissolved in DMSO) for 4 h and then BCAA was added for an additional 24 h of culture. The control group was cultured in medium containing 0.1% DMSO for 24 h.

### Lentiviral transduction

The shPPM1K-mCherry lentivirus targeting the mouse PPM1K was prepared by OBiO Technology Corp.,Ltd (Shanghai, China). Lentivirus expressing scramble shRNA (vector) was used as a negative control. For lentivirus transduction, primary cortical neurons were seeded on the poly-L-lysine coated plates or dishes as above. Three days postseeding, the medium was removed and added the fresh medium containing shPPM1K or vector lentivirus at a certain multiplicity of infection (MOI). Twenty-four hours post-transduction, the medium containing lentivirus was replaced with a fresh culture medium. Transduced neurons were incubated for 3 days and then harvested to detect the transduction efficiency. The efficiency of Lenti-shPPM1K was validated by qRT-PCR and Western blotting. Finally, based on the pre-experimental results, an MOI of 20 was selected to continue transduction and used for subsequent observation and analysis. The sequences of shRNA were provided in Table S2.

### Cell viability and lactate dehydrogenase (LDH) assay

Cells were seeded onto 96-well plates (3 × 10^4^ cells per well), and their viability was assessed after treatment using a Cell Counting Kit-8 (CCK-8) assay (IC1519, InCellGene, USA). Briefly, 10 μL of reagent was incubated with cells in a 96-well plate at 37 °C for 1 h and then the absorbance was measured at 450 nm by a multimode microplate reader (Tecan Infinite M200).

Cells were seeded onto six-well plates at a density of 5 × 10^5^ cells/well, and then the cellular supernatants were collected at the assay endpoint to detect LDH levels (A020-2-2, Nanjing Jiancheng). The absorbance of each well at 450 nm was measured with a multimode microplate reader (Tecan Infinite M200).

### Immunocytochemistry

Cells were grown on cell glass slides, rinsed with PBS, fixed with 4% PFA, and washed with PBS again. Next, the slides were blocked with 3% BSA with 0.3% Triton X-100 for 1 h, and incubated with the following primary antibodies overnight at 4 °C: ACSL4, 4-HNE, NeuN (ABN78, 1:1000, Sigma-Aldrich), and beta III tubulin (Tuj1; ab78078, 1:500, Abcam). After washing slides with PBS three times, they were incubated with corresponding secondary antibodies and DAPI in the dark. Finally, slides were mounted with glycerol and imaged using a confocal microscope (Olympus FV 3000).

### Measurement of lipid peroxidation with C11-BODIPY 581/591 staining

Following all interventions, cells grown in confocal dishes were stained with 2 μM C11-BODIPY 581/591 (D3861, Invitrogen, Carlsbad, CA, USA) for 30 min at 37 °C. Lipid ROS levels in primary neuronal cells were analyzed using a confocal microscope (Olympus FV 3000). Nonoxidized C11 was observed under the 594-nm excitation channel, while oxidized C11 was observed under the 488-nm excitation channel. Lipid ROS levels were assessed as the mean fluorescence intensity (MFI) of the 488-nm excitation channel.

### Monitoring on cerebral blood flow

Cerebral blood flow (CBF) of each group was monitored 24 h after cerebral I/R, using a laser speckle Doppler flowmeter (RFLSI III, RWD Life Science Co, Shenzhen, China). The images and data analysis were processed with the RFLSI analysis software (RWD Life Science). The change in CBF was quantitated and expressed as a relative blood flow that was calculated as the ratio of the region of interest (ROI) areas between the ipsilateral and contralateral hemispheres [23].

### Assessment of neurological deficits

At 1, 3, 5, 7 and 14 days after reperfusion, the neurological functions of mice in each group were evaluated according to Longa scores and the Garcia scale [24], which are widely used for behavioral assessment of mice after focal cerebral ischemia [25]. Assessments were performed by a blinded observer and then confirmed by another blinded observer.

### CatWalk XT gait test

To assess functional impairment and recovery, mice underwent a CatWalk XT automated quantitative gait analysis (CatWalk XT; Noldus, Wageningen, Netherlands) at 1, 3, 5, 7, and 14 days after I/R, and were trained 3 days before the experiment as previously described [26, 27]. Briefly, mouse paw prints were captured by a high-speed video camera positioned underneath a long narrow walkway when the mouse passed. Mice were put on one end of the walkway and crossed it to reach a dark chamber that led to the animal’s home cage. Three consecutive trials were performed for each animal. Images from each trial were digitized and analyzed by Catwalk-XT software (Noldus).

### 2,3,5-Triphenyltetrazolium chloride (TTC) staining

After reperfusion for 24 h, mice were anesthetized and their fresh brains were removed. Subsequently, whole-brain tissue was evenly cut into 1-mm coronal sections with a metallic brain matrix, and the resulting sections were incubated in 1% TTC (T8877, Sigma-Aldrich) for 30 min at 37 °C in the dark. Subsequently, sections were fixed with 4% PFA and stored until imaging. The percentage of infarct volume was calculated according to the formula: cerebral infarction volume/total cerebral volume × 100%.

### Statistics

All statistical analyses were performed using GraphPad Prism 9 software (GraphPad Software, San Diego, CA, USA). Data are presented as the mean ± standard deviation (SD). Independent sample two-tailed Student’s *t*-test and Wilcoxon tests were used to analyze the two groups. For multiple comparisons, one-way ANOVA was performed, followed by Tukey’s post hoc test. Assessment of neurological scores and gait test results were analyzed by two-way repeated measures ANOVA. Cumulative survival rates were compared among the four groups using the Kaplan–Meier method and log-rank tests. All experiments were performed at least three independent times. A value of *P* < 0.05 was used to indicate statistical significance.

### Further materials and methods

The other detailed materials and methods and full and uncropped western blots are presented in the supplementary materials.

## Results

### Brain metabolic profiles revealed differential metabolic models after acute cerebral ischemic

**Fig. 1:**
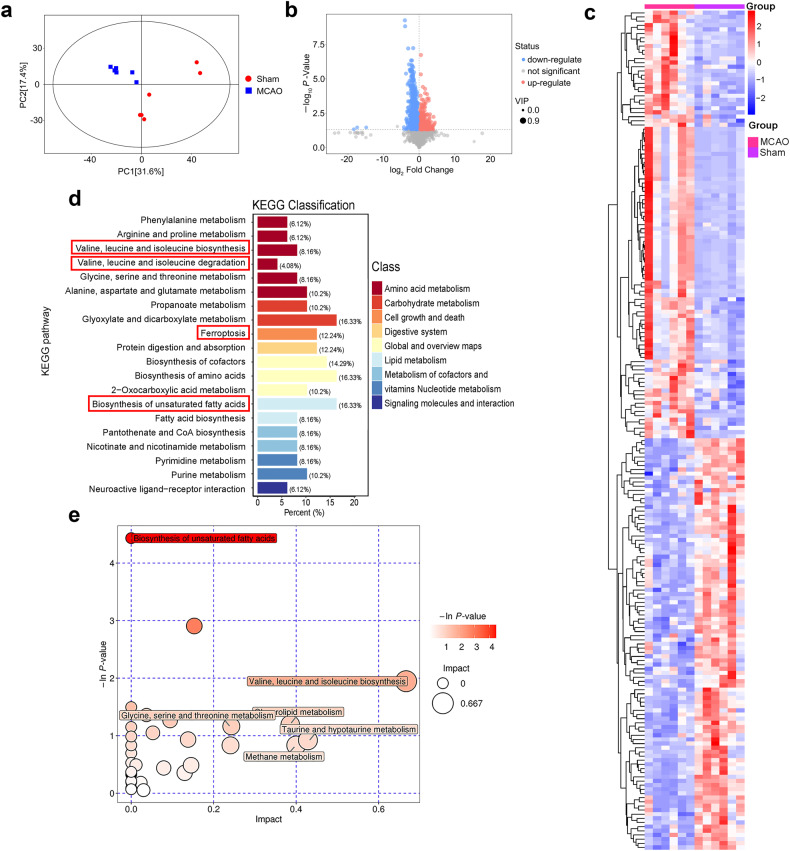
Diversity and abundance analysis based on untargeted LC-MS and GC-MS analysis. **a** Principal component analysis of MCAO and sham groups. **b** Volcano plot comparing MCAO vs. sham groups; blue: downregulated, red: upregulated in MCAO group. **c** Heatmap of differentially accumulated metabolites in MCAO vs. sham groups. **d** Pathways enriched for metabolic variations after MCAO. **e** Kyoto Encyclopedia of Genes and Genomes pathway bubble chart of differentially accumulated metabolites for MCAO vs sham groups. GC-MS gas chromatography-mass spectrometry, LC-MS liquid chromatography-mass spectrometry, MCAO middle cerebral artery occlusion.

To investigate molecular changes occurring after acute cerebral ischemia, we performed metabolite detection to explore candidate molecular players that might exert pivotal roles. Principal component analysis (PCA) resulted in obvious clustering of the samples (Fig. 1a), which revealed differentially expressed metabolic profiles between sham and MCAO groups. Based on metabolome data detected by both GC-MS and LC-MS platforms, metabolites with *P* < 0.05 and values of variable importance in the project > 1 calculated by orthogonal partial least-squared discriminant analysis were identified as differential metabolites. The resulting differential metabolites were visualized in a volcano plot and heatmap of hierarchical clustering (Fig. 1b, c). A total of 202 differential metabolites are shown, of which 103 were upregulated and 99 were downregulated in the MCAO group compared with the sham group. In general, changes of these important metabolites in the MCAO group were quite different from those in the sham group.

To further explore its potential mechanisms in cerebral ischemia, we performed metabolic pathway analysis of differential metabolites using the Kyoto Encyclopedia of Genes and Genomes (KEGG) metabolic library. The results indicate significant differences in metabolic pathways related to amino acids and lipid fatty acids (Fig. 1d, e). Differential amino acids are shown in Supplementary Table S3, and the top ten differentially expressed fatty acids are shown in Supplementary Table S4. We found that the amino acids valine and isoleucine were elevated in the MCAO group, while unsaturated fatty acids, such as arachidonic acid and gamma-linolenic acid, were decreased. Accordingly, we focused on “Biosynthesis of unsaturated fatty acids”, “Valine, leucine and isoleucine biosynthesis”, and “Ferroptosis” pathways. Arachidonic acid is associated with the ferroptosis pathway (Fig. S1). Metabolomics results indicate that BCAA and PUFA metabolism were prominently abnormal, while PUFA was closely associated with ferroptosis, indicating a correlation between BCAA and ferroptosis.

### BCAA levels and neuronal ferroptosis were increased 24 h after cerebral I/R

**Fig. 2:**
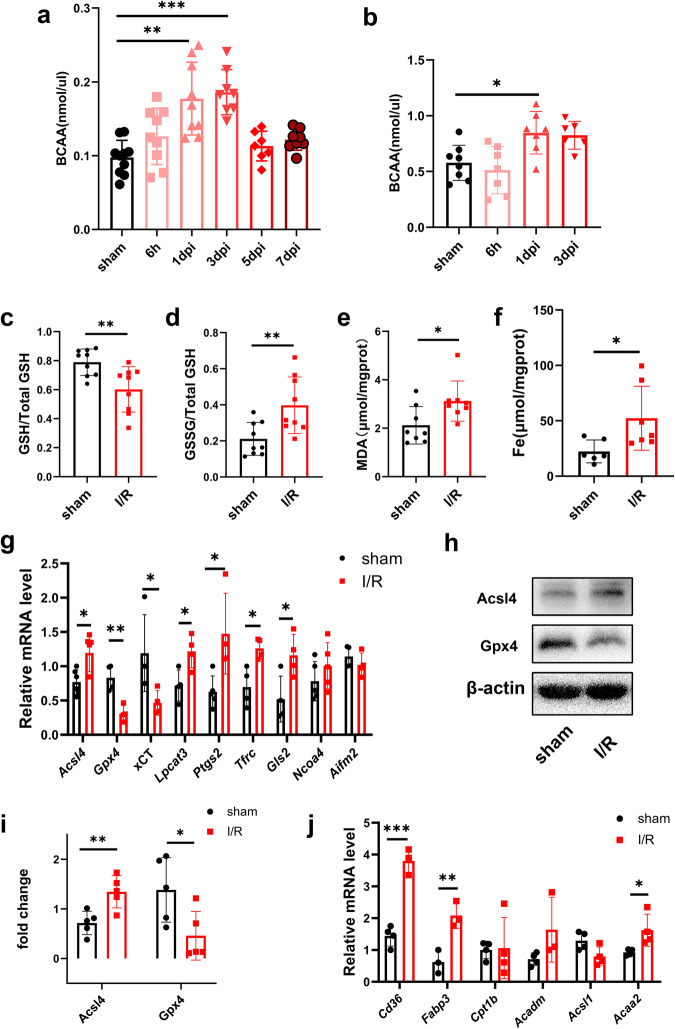
BCAA levels and neuronal ferroptosis were elevated in mice 24 h after cerebral I/R. **a** BCAA levels in sham and I/R group tissues at 6 h and 1, 3, 5, and 7 days after reperfusion (*n* = 7–10/group, one-way ANOVA followed by multiple comparisons). **b** Serum BCAA levels of sham and I/R groups at 6 h, and 1 and 3 days after reperfusion (*n* = 6–8/group, one-way ANOVA followed by multiple comparisons). **c**–**f** Quantification of tissue levels of GSH (**c**) (n = 9/group, *t* test), GSSG (**d**) (*n* = 9/group, *t* test), MDA (**e**) (*n* = 8/group, *t* test), and iron (**f**) (*n* = 6–7/group, *t* test) at 1 day in sham and I/R groups. **g** mRNA expression of ferroptosis marker genes *Acsl4*, *Gpx4*, xCT, *Lpcat3*, *Ptgs2*, *Tfrc*, *Gls2*, *Ncoa4*, and *Aifm2* at 24 h post cerebral I/R in sham and I/R groups (*n* = 4–6/group, *t* test). **h**, **i** Quantification of Acsl4 and Gpx4 protein expression levels in sham and I/R groups. β-actin was used as a loading control (*n* = 5/group, *t* test). **j** mRNA expression of FAO-related genes *Cd36*, *Fabp3*, *Cpt1b*, *Acadm*, *Acsl1*, and *Acaa2* at 1 day post cerebral I/R in sham and I/R groups (*n* = 3–4/group, *t* test). All data are presented as mean ± SD, *P < 0.05, **P < 0.01, ***P < 0.001.

Considering the relationship between lipid peroxidation and cellular ferroptosis [28], as well as our metabolomics data for BCAA and lipid peroxidation, we next explored the role of BCAA in ferroptosis after cerebral I/R injury. Firstly, we examined changes of BCAA in a mouse cerebral I/R model, the data show that the BCAA level in brain tissue was increased at 6 h, peaked at 3 days, and gradually decreased at 5 and 7 days after cerebral I/R injury (Fig. 2a). In addition, compared with the sham group, serum BCAA levels were significantly increased 24 h after cerebral I/R (Fig. 2b). We also measured several representative biomarkers of oxidation and lipid peroxidation after cerebral I/R, including GSH, 4-HNE, and MDA [29]. The results show that GSH was decreased (Fig. 2c) and levels of GSSG (Fig. 2d) and MDA (Fig. 2e) were increased in the cerebral I/R group. We further detected ferroptosis at 24 h after cerebral I/R, the results show that iron levels were higher (Fig. 2f) and mRNA levels of ferroptosis-related genes *Acsl4*, *Lpcat3*, *Ptgs2*, *Tfrc*, and *Gls2* were significantly increased after cerebral I/R, while those of *Gpx4* and xCT were decreased (Fig. 2g). However, mRNA levels of *Ncoa4* and *Aifm2* were not significantly different between the two groups (Fig. 2g). Likewise, protein levels of Acsl4 increased and Gpx4 decreased in the cerebral I/R group (Fig. 2h, i). In addition, we examined the expression of fatty acid oxidation (FAO)-related genes. mRNA levels of genes encoding FAO-related enzymes involved in fatty acid transport (*Cd36* and *Fabp3*) and β-oxidation (*Acaa2*) were increased 24 h after cerebral I/R, while there were no differences in *Acsl1*, *Cpt1b*, or *Acadm* levels (Fig. 2j).

**Fig. 3:**
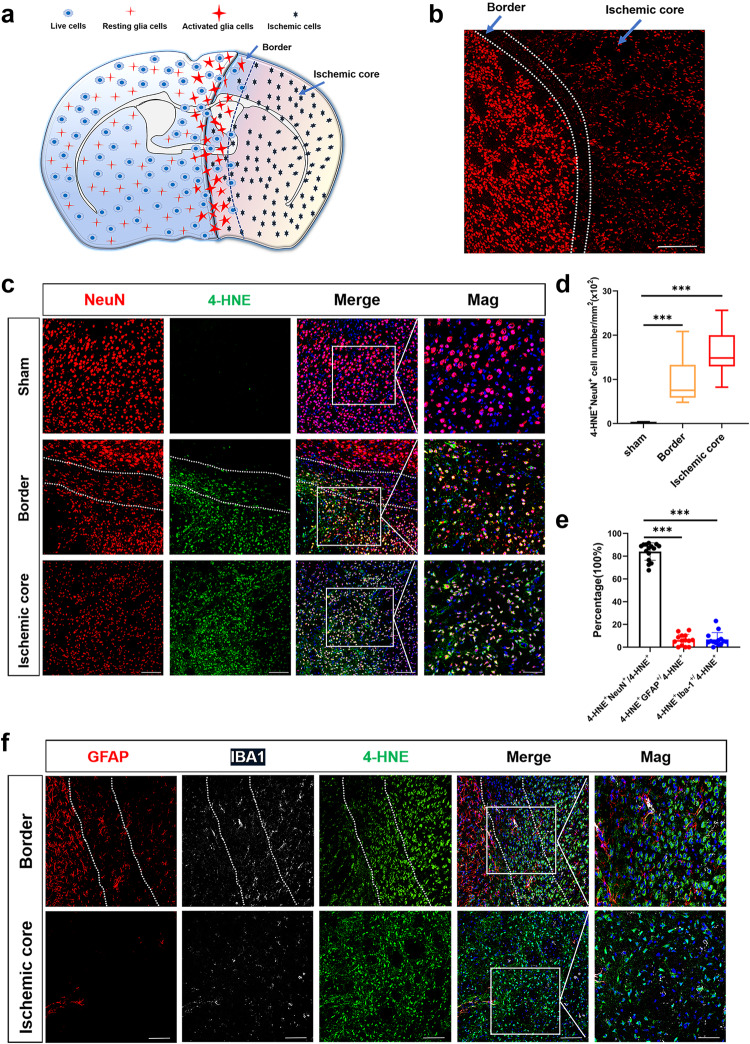
Spatiotemporal patterns of ferroptosis 24 h after cerebral I/R. **a** Schematic showing the distribution of various cell types after cerebral I/R. **b** Immunofluorescence staining for NeuN reveals the ischemic core and ischemic border areas. Scale bar = 200 μm. **c** Immunofluorescence staining for NeuN and 4-HNE at 24 h postcerebral I/R in sham and I/R groups, including ischemic border (between the two dotted lines) and ischemic core areas. Scale bar = 100 μm. Representative magnified images are on the far right. Scale bar = 50 μm. **d** Quantification of NeuN-positive and 4-HNE-positive cells (*n* = 16–17 images from four animals/group, one-way ANOVA followed by multiple comparisons). **e**, **f** Representative images of immunostaining of 4-HNE with GFAP and IBA1 (white), and quantification of percentages of 4-HNE/NeuN double-positive cells, 4-HNE/GFAP double-positive cells and 4-HNE/IBA1 double-positive cells in the ischemic border (between the two dotted lines) and ischemic core area at 1 day post cerebral I/R (*n* = 14–15 images from four animals/group, one-way ANOVA followed by multiple comparisons). Scale bar = 100 μm. Representative magnified images are on the far right. Scale bar = 50 μm. All data are presented as mean ± SD, ***P < 0.001.

We further examined the identity of cells undergoing ferroptosis 24 h after cerebral I/R by performing immunofluorescence (IF) analysis in mice. IF staining of NeuN showed smaller and irregular NeuN-positive fluorescence signals in the ischemic area after I/R. We observed a band between ischemic and normal tissue (Fig. 3a, b) that was defined as the ischemic border according to previous studies [20, 23]. Double staining of 4-HNE and various cell markers was further performed to observe the spatial distribution and pattern of ferroptosis. The results demonstrate that the expression of 4-HNE mainly occurred in NeuN-positive cells. Quantitative analysis revealed that numbers of both 4-HNE- and NeuN-positive cells were significantly increased in the ischemic border and ischemic core areas after cerebral I/R (Fig. 3c, d). IF analysis demonstrated that approximately 84% of 4-HNE-positive cells were NeuN-positive, while 6.3% were GFAP-positive and 6.8% were IBA1-positive (Fig. 3e, f). These results further indicate a relationship between BCAA and neuronal lipid peroxidation after cerebral I/R.

### BCAA induced neuronal ferroptosis

**Fig. 4:**
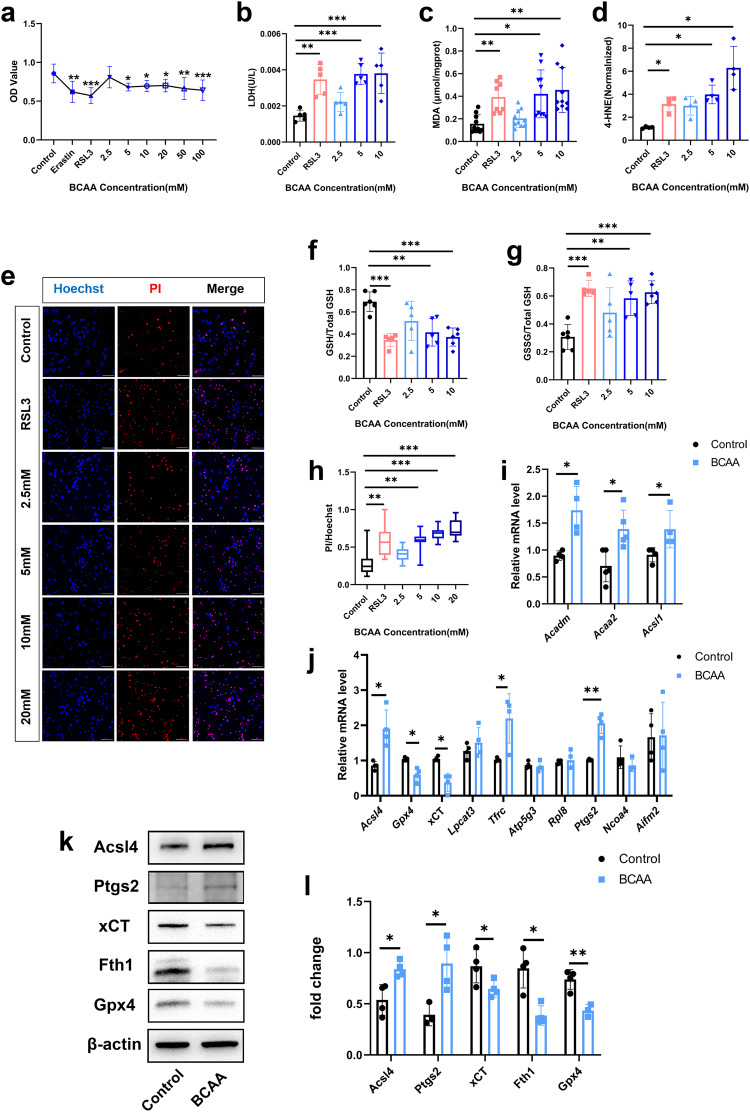
BCAA induced neuronal ferroptosis. **a** Cell viability of neurons was detected by CCK-8 assay following incubation with erastin (10 μM) and RSL3 (5 μM) for 2 h, or 2.5, 5, 10, 20, 50, and 100 mM BCAA for 24 h. **b**–**g** Neurons were treated with RSL3 (5 μM) for 2 h and 2.5, 5, 10, or 20 mM BCAA for 24 h. LDH release analysis of neurons (**b**). MDA (**c**), 4-HNE (**d**), GSH (**f**), and GSSG (**g**) concentrations in neurons, as detected by ELISA. **e** PI-Hoechst double-stained neurons treated with RSL3 and varying BCAA concentrations. **h** Quantification of PI-positive/Hoechst double-positive cells. Scale bar = 50μm. **i** Expression of fatty acid oxidation (FAO)-associated mRNA transcripts of *Acadm*, *Acaa2*, and *Acsl1* in neurons treated with medium and BCAA (5 mM) after 24 h. **j** mRNA expression of ferroptosis marker genes *Acsl4*, *Gpx4*, xCT, *Lpcat3*, *Tfrc*, *Atp5g3*, *Rpl8*, *Ptgs2*, *Ncoa4*, and *Aifm2* in neurons treated with medium and BCAA (5 mM) after 24 h. **k**, **l** Representative immunoblot and statistical analysis of ferroptosis marker proteins Acsl4, Ptgs2, xCT, Fth1, and Gpx4 from neurons under normal or BCAA (5 mM)-treated conditions. All experiments were performed at least three independent times. All data are presented as mean ± SD, *P < 0.05, **P < 0.01, ***P < 0.001.

To further definite the role of BCAA in neuronal ferroptosis, we first investigated whether BCAA could induce neuronal lipid peroxidation that resulted in ferroptosis in vitro. We treated primary cortical neurons with the ferroptosis inducer erastin, RSL3, and varying concentrations of BCAA. As shown in Fig. 4a, we found that except for in the presence of erastin, RSL3 could significantly decrease cell viability and BCAA at 5 mM and above conferred a significant and dose-dependent decrease in cell viability. Similarly, PI/Hoechst double-staining results show that RSL3 and BCAA induced cell death in a dose-dependent manner (Fig. 4e–h). Further, LDH release assay results confirmed that RSL3 and 5–10 mM BCAA significantly induced cell damage (Fig. 4b). IF analysis showed that only 5–10 mM BCAA could significantly increase the Acsl4 level (Fig. S2a, b). We found that 5–10 mM BCAA depleted GSH and caused accumulation of GSSG, MDA and 4-HNE (Fig. 4c–g). In Li’s study of myocardial I/R, BCAA increased the susceptibility of myocardial cells to oxygen glucose deprivation/re-oxygenation injury by enhancing FAO [30]. Our results show that mRNA levels of FAO-related genes *Acadm*, *Acaa2*, and *Acsl1* were significantly increased (Fig. 4i), as were levels of ferroptosis-related genes *Acsl4*, *Tfrc*, and *Ptgs2*; whereas, *Gpx4* and xCT levels were significantly decreased following BCAA exposure. There were no significant differences in expression levels of *Lpcat3*, *Atp5g3*, *Rpl8*, *Ncoa4*, or *Aifm2* (Fig. 4j). Protein levels of Acsl4 and Ptgs2 were significantly increased, and protein levels of xCT, Fth1, and Gpx4 were significantly decreased after BCAA exposure (Fig. 4k, l). We further detected the intracellular Fe^2+^, the results showed that there was no significant change between the control and BCAA groups (Fig. S2d, e). In addition, except for FTH1, iron transport-related proteins Fpn and Tfrc also have no significant changes between the two groups (Fig. S2f, g). These results show direct evidence linking BCAA and neuronal ferroptosis, and the BCAA-induced ferroptosis mainly by affecting lipid oxidation and antioxidant system.

**Fig. 5:**
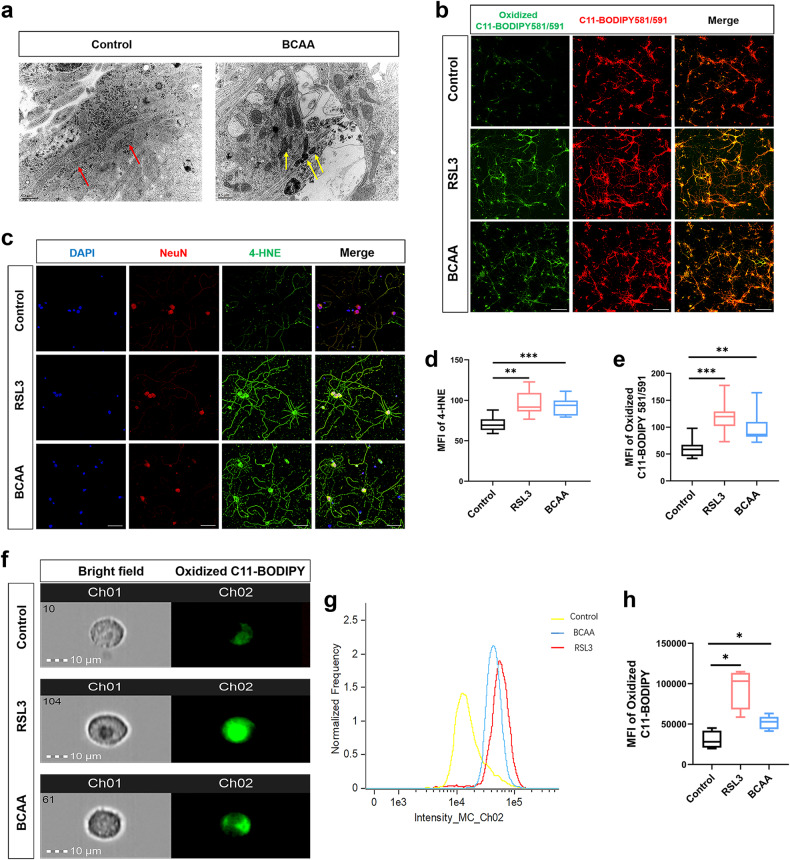
Neuronal ultrastructural changes and increased lipid peroxidation marked the occurrence of ferroptosis after BCAA exposure. **a** Electron microscopy images of neurons under normal or BCAA (5 mM)-treated conditions at 24 h. Red arrows indicate normal mitochondria and yellow arrows indicate abnormal mitochondria with increased membrane density, shrinkage, and cristae disorder. Scale bar = 0.5 μm. **b, e** Lipid peroxidation levels of neurons stained with C11-BODIPY581/591 to monitor oxidized and non-oxidized variants of lipid peroxides. **b** Representative images of C11-BODIPY 581/591 staining among control, RSL3, and BCAA groups, and quantification of mean immunofluorescence intensities (MFI) of oxidized C11-BODIPY 581/591 (**e**). **c**, **d** Representative images of 4-HNE and NeuN double-stained neurons, and quantitative analysis of 4-HNE MFI in each group of neurons. **f** Representative images of cells following incubation with the fluorescent probe C11-BODIPY 581/591 show the presence of oxidized variants of lipid peroxides signals (Ch02) at 40× magnification, Ch01 = bright field. Scale bar = 10 μm. **g** Single-color histogram of Ch02 fluorescence intensity. All images are representative and were chosen at random. **h** Quantification of mean intensities of Ch02. All experiments were performed at least three independent times. All data are presented as mean ± SD, *P < 0.05, **P < 0.01, ***P < 0.001.

To further confirm the occurrence of neuronal ferroptosis upon BCAA exposure, we observed the ultrastructural changes of neurons. The results revealed increased mitochondrial membrane density, mitochondrial shrinkage, and mitochondrial cristae disorder in BCAA-treated neurons (Fig. 5a), which are all characteristics of ferroptosis [4]. IF results show that 4-HNE expression was significantly increased in neurons in RSL3- and BCAA-treated groups (Fig. 5c, d). Because the accumulation of lipid peroxides is an intrinsic quality of ferroptosis, we further detected lipid ROS levels. RSL3 and BCAA increased lipid ROS levels (Fig. 5b, e). Imaging flow cytometry results of BCAA-treated SH-SY5Y cells further demonstrated that BCAA increased neuronal lipid ROS (Fig. 5f–h). Altogether, these data indicate that BCAA induced lipid peroxidation and ferroptosis in neurons.

### Metabolic dysfunction of neuronal BCAA increased BCAA levels after cerebral I/R

**Fig. 6:**
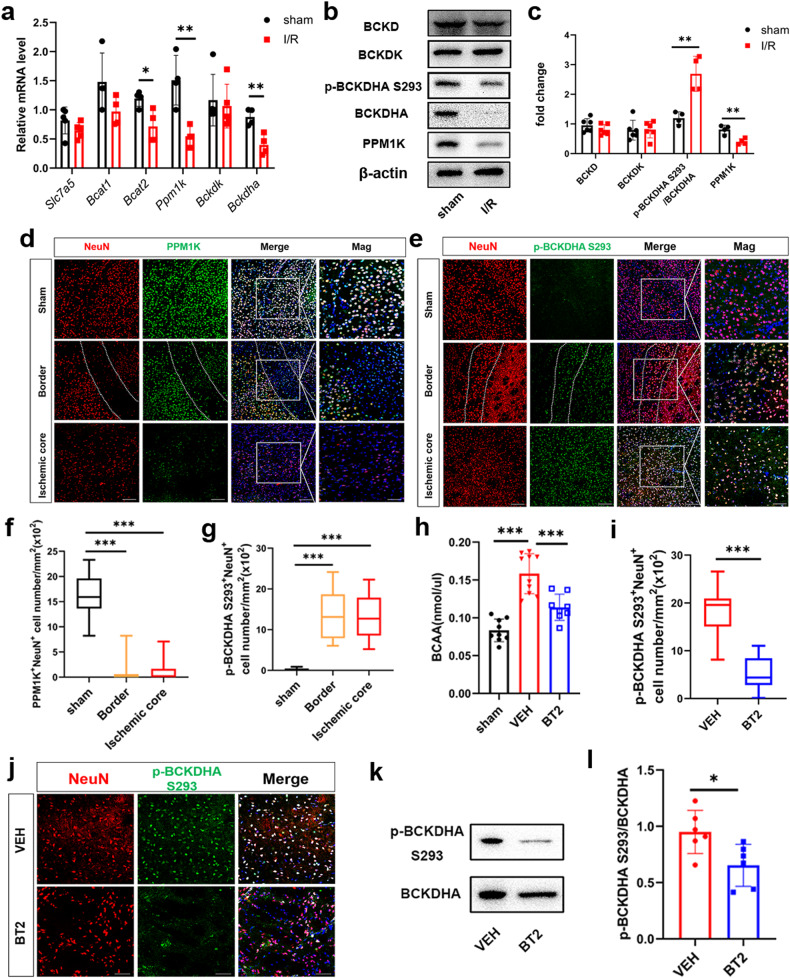
Metabolic dysfunction of neuronal PPM1K and BCKDHA increased BCAA levels 24 h after cerebral I/R. **a** mRNA expression of *Slc7a5*, *Bcat1*, *Bcat2*, *Ppm1k*, *Bckdk* and *Bckdha* at 24 h post cerebral I/R (*n* = 4–5/group, *t* test). **b** The representative picture of immunoblots for BCKD, BCKDK, p-BCKDHA S293, BCKDHA, PPM1K and β-actin at 24 h postcerebral I/R. **c** Quantification analysis for the immunoblots in (**b**) (*n* = 4–6/group, *t* test). **d** Representative images of NeuN/PPM1K staining at 24 h post cerebral I/R in sham and I/R groups, including ischemic border (between the two dotted lines) and ischemic core areas. Scale bar = 100 μm. Representative magnified images are on the far right. Scale bar = 50 μm. **f** Quantification of NeuN-positive and PPM1K-positive cells number (*n* = 15–18 images from four animals/group, one-way ANOVA followed by multiple comparisons). **e** Immunofluorescence staining for NeuN/p-BCKDHA S293 at 24 h postcerebral I/R in sham and I/R groups, including ischemic border (between the two dotted lines) and ischemic core areas. Scale bar = 100 μm. Representative magnified images are on the far right. Scale bar = 50 μm. **g** Quantification of NeuN-positive and p-BCKDHA S293-positive cells (*n* = 12-18 images from four animals/group, one-way ANOVA followed by multiple comparisons). **h** BT2 decreased BCAA levels in tissue compared with the VEH group 24 h after reperfusion (*n* = 8–10/group, one-way ANOVA followed by multiple comparisons). **i** Quantification of NeuN-positive and p-BCKDHA S293-positive cells (*n* = 18–21 images from four animals/group, *t* test). **j** Immunofluorescence staining of NeuN/p-BCKDHA S293/DAPI in the ischemic core area 24 h postcerebral I/R in VEH and BT2 groups. Scale bar = 50μm. **k** Representative bands of p-BCKDHA S293 and BCKDHA. **l** Quantitative analysis of p-BCKDHA S293/BCKDHA protein expression (*n* = 6/group, *t* test). All data are presented as mean ± SD, *P < 0.05, **P < 0.01, ***P < 0.001.

To explore the mechanism of BCAA elevation, we detected BCAA metabolism-related enzymes. qRT-PCR results show that mRNA expression of *Slc7a5* (BCAA transporter) and *Bcat1* was slightly decreased, while *Bcat2* mRNA expression was significantly decreased (Fig. 6a). Branched-chain aminotransferase (BCAT) reversibly inverts the BCAA to its corresponding BCKA [31]. mRNA levels of *Ppm1k* and *Bckdha* were significantly decreased after cerebral I/R, while no significant difference in *Bckdk* (Fig. 6a). BCKA dehydrogenase (BCKD) complex irreversibly oxidizes BCKA [32]. The phosphorylation state of the BCKD complex subunit E1α (BCKDHA Ser293) determines its activity, which can be hyper-phosphorylated and inactivated through BCKD kinase (BCKDK). Conversely, PPM1K specifically induces dephosphorylation and activation of BCKDHA Ser293 [31, 33]. Similarly, protein levels of BCKD and BCKDK were not significantly changed, PPM1K was markedly decreased, and the phosphorylation level of BCKDHA S293 was significantly increased (Fig. 6b, c).

To validate the potential mechanism, we performed IF analysis. The results of PPM1K/NeuN show that numbers of PPM1K-positive and NeuN-positive cells were significantly decreased in neurons in the ischemic border and ischemic core areas at 24 h after I/R injury (Fig. 6d, f). In contrast, numbers of double positive cells of p-BCKDHA S293 and NeuN were significantly increased (Fig. 6e, g). Taken together, these results suggest that the decrease in PPM1K might mediate BCAA elevation by phosphorylating BCKDHA S293 to disrupt oxidative pathways.

### BT2 improved BCAA metabolism in cerebral I/R

To further verify the involvement of BCAA in the oxidative metabolic disorder induced by elevated p-BCKDHA S293 after cerebral I/R, the BCKDK inhibitor BT2 was applied, which can effectively activate BCKDH in various tissues [16]. Notably, compared with the VEH group, BCAA levels were dramatically decreased after BT2 treatment (Fig. 6h). Immunofluorescence staining revealed an obvious reduction in NeuN/p-BCKDHA S293 double-positive cells in the ischemic core area after BT2 treatment (Fig. 6i, j). Meanwhile, the protein level of BCKDHA S293 phosphorylation was remarkably reduced in the BT2 group 24 h after cerebral I/R (Fig. 6k, l). Additionally, our results suggest that BCAA did not cause changes in BCAA metabolizing enzymes (Fig. S2c–e). Collectively, these results confirm that PPM1K mediated-BCKDHA S293 phosphorylation contributed to BCAA metabolic disorder 24 h after cerebral I/R and could be ameliorated by BT2.

### BT2, Fer-1, and PPM1K knockdown inhibited neuronal ferroptosis induced by BCAA and cerebral I/R

**Fig. 7:**
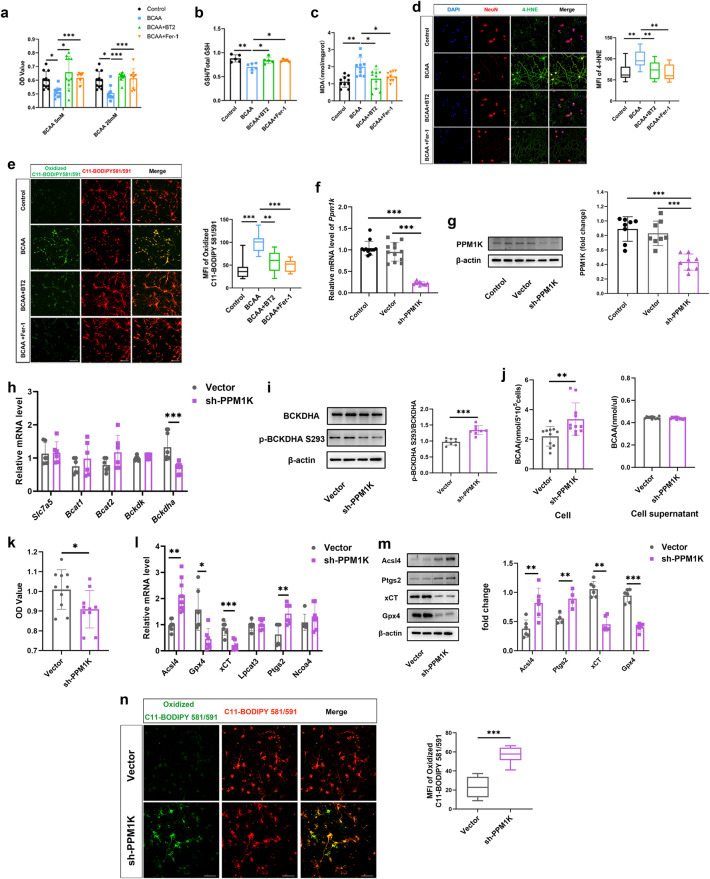
BT2, Fer-1, and PPM1K knockdown mitigated BCAA induced neuronal ferroptosis. **a**–**e** Neurons were pretreated with BT2 (80 μM) or Fer-1 (5 μM) for 4 h and then incubated with BCAA for 24 h. **a** CCK-8 assay of neurons showed the BT2 and Fer-1 rescued BCAA (5, 20 mM)-induced reductions in cell viability. GSH (**b**) and MDA (**c**) levels in neurons were detected by ELISA among control, BCAA (5 mM), BT2 + BCAA, and Fer-1 + BCAA groups. Staining for 4-HNE and C11-BODIPY581/591 shows that BT2 and Fer-1 decreased lipid peroxidation levels. **d** Representative images of 4-HNE/NeuN double staining in each group of neurons and quantification analysis of 4-HNE MFI. **e** Representative images of C11-Bodipy581/591 staining in each group of neurons and quantification MFI of oxidized C11-Bodipy581/591. **f** mRNA expression of PPM1K in neurons treated with medium, infected with Lenti-vector, or Lenti-shPPM1K. (**g**) Representative immunoblot and statistical analysis of PPM1K in neurons treated medium, Lenti-vector, or Lenti-shPPM1K. **h**–**n** Neurons were treated with Lenti-vector or Lenti-shPPM1K. **h** mRNA expression of *Slc7a5*, *Bcat1*, *Bcat2*, *Ppm1k*, *Bckdk* and *Bckdha* in neurons. **i** The representative picture of immunoblots and quantification analysis for p-BCKDHA S293/BCKDHA. **j** BCAA levels in neurons and neurons supernatant. **k** Neurons viability was detected by CCK-8 assay. **l** mRNA expression of ferroptosis marker genes *Acsl4*, *Gpx4*, xCT, *Lpcat3*, *Ptgs2*, and *Ncoa4* in neurons. **m** Representative immunoblot and statistical analysis of ferroptosis marker proteins Acsl4, Ptgs2, xCT, and Gpx4 in neurons. **n** Representative images of C11-Bodipy581/591 staining and quantification MFI of oxidized C11-Bodipy581/591. Scale bar = 50 μm. All experiments of neurons were performed at least three independent times. All data are presented as mean ± SD, *P < 0.05, **P < 0.01, ***P < 0.001.

To further explore the influence of BCAA on neuronal ferroptosis after cerebral I/R, we used Fer-1, a reported ferroptosis inhibitor [28], and BT2 to test whether they could rescue the neuronal injury induced by BCAA. First, 10–160 μM BT2 and 2–10 μM Fer-1 had no significant effect on neuronal activity (Fig. S3a, b). We chose 80 μM BT2 and 5 μM Fer-1 for subsequent experiments. We found that pretreatment with BT2 and Fer-1 significantly alleviated the decline of neuronal activity induced by 5 mM and 20 mM BCAA (Fig. 7a). PI/Hoechst double staining shows that BT2 and Fer-1 could alleviate BCAA-induced neuronal death (Fig. S3c, d). Our results also show that BT2 and Fer-1 markedly diminished BCAA-induced GSH depletion, as well as MDA accumulation (Fig. 7b, c). In addition, BT2 and Fer-1 decreased levels of lipid peroxidation and lipid ROS levels induced by BCAA (Fig. 7d, e). These results demonstrate that BT2 and Fer-1 significantly inhibited neuronal ferroptosis induced by BCAA.

We further found that defective BCAA catabolism could induce neuronal ferroptosis by PPM1K knockdown. Neurons were transfected with medium (control), lentivirus vectors (Lenti-vector), or vectors carrying PPM1K shRNA (Lenti-shPPM1K). The effect and efficiency of knocking down of Lenti-shPPM1K was verified by qRT-PCR and western blotting (Fig. 7f, g). Compared with the Lenti-vector group, PPM1K knockdown induced BCAA catabolic defects, decreased mRNA level of *Bckdha*, and increased p-BCKDHA S293 expression and BCAA level in neurons (Fig. 7h-j). Furthermore, in PPM1K knockdown neurons, the cell viability, mRNA levels of *Gpx4* and xCT were significantly decreased (Fig. 7k, l), while mRNA levels of *Acsl4* and *Ptgs2* were significantly increased (Fig. 7l). There were no significant differences in expression levels of *Lpcat3* or *Ncoa4* (Fig. 7l). Protein levels of Acsl4 and Ptgs2 were significantly increased, and protein levels of xCT and Gpx4 were significantly decreased after PPM1K knockdown (Fig. 7m). In addition, the lipid ROS level was also increased significantly in Lenti-shPPM1K group (Fig. 7n). Taken together, these data demonstrate that defective BCAA catabolism induced neuronal ferroptosis via downregulating PPM1K expression.

### BT2 and Fer-1 alleviated neuronal ferroptosis, CBF reduction, tissue damage and promoted neurological functional recovery after cerebral I/R injury

**Fig. 8:**
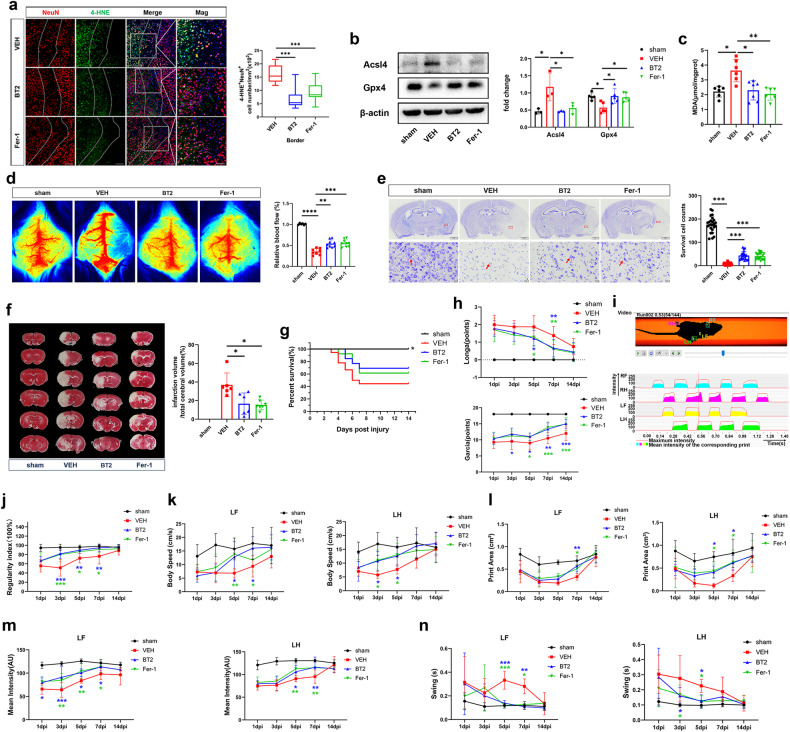
BT2 and Fer-1 protected against neuronal ferroptosis, cerebral blood flow reduction, tissue damage, and promoted neurological functional recovery after cerebral I/R. **a** Representative images of NeuN/4-HNE staining and quantitative analysis of p-BCKDHA S293-positive and 4-HNE-positive cells in the ischemic border among VEH, BT2, and Fer-1 groups (*n* = 14–18images from four animals/group, one-way ANOVA followed by multiple comparisons). Scale bar = 100 μm. Representative magnified images are shown on the far right. Scale bar = 50 μm. **b** Representative western blots and quantitative analysis for of Acsl4 and Gpx4 protein expression, normalized to β-actin (*n* = 3–5/group, one-way ANOVA followed by multiple comparisons). **c** Quantification shows BT2 and Fer-1 significantly reduced brain tissue levels of MDA (*n* = 6–7/group, one-way ANOVA followed by multiple comparisons). **d** Laser speckle imaging 24 h after cerebral I/R and quantification of relative blood flow. (*n* = 6–9/group, one-way ANOVA followed by multiple comparisons). **e** Representative images of Nissl staining and quantification of surviving cells among sham, VEH, BT2, and Fer-1 groups. Scale bar = 1 mm in the upper panel and scale bar = 20 μm in the lower panel (*n* = 21–25 images from three animals/group, one-way ANOVA followed by multiple comparisons). **f** Representative images of TTC staining and qquantitative analysis of the percentage of infarct volume relative to total cerebral volume among sham, VEH, BT2, and Fer-1 groups 24 h after cerebral I/R (*n* = 6–7/group, one-way ANOVA followed by multiple comparisons). **g** Survival curves of sham, VEH, BT2, and Fer-1 groups during the 14-day observation period. Mortality data for the sham group was significantly differences from the other groups, *P < 0.05 as determined by Log-rank (Mantel-Cox) test. **h** Neurological changes were observed by Longa neurological scores (upper) and Garcia neurological scores (lower) among sham, VEH, BT2, and Fer-1 groups from 3–14 days. **i** A diagrammatic sketch marked with footprints and footprint intensity of the sham group. BT2 and Fer-1 significantly increased body regularity index (**j**), body speed (**k**), print area (**l**), mean intensity (**m**), and swing time (**n**) of the left forepaw and left hindpaw compared with the VEH group. *n* = 8–10/group, two-way ANOVA (Bonferroni’s multiple comparison test). LF: left forepaw, LH: left hindpaw. All data are presented as mean ± SD, *P < 0.05, **P < 0.01, ***P < 0.001.

Next, we tested whether BT2 or Fer-1 could rescue the neuronal injury induced by BCAA. Double staining of 4-HNE and NeuN displayed high numbers of 4-HNE- and NeuN-positive cells in the ischemic border and ischemic core areas of VEH group mice. BT2 and Fer-1 obviously decreased 4-HNE expression (Fig. 8a and Fig. S4a, b). Western blotting further showed that BT2 and Fer-1 significantly inhibited the increase in Acsl4 and decrease in Gpx4 induced by cerebral I/R (Fig. 8b). In addition, BT2 and Fer-1 significantly alleviated levels of the lipid peroxidation product MDA after cerebral I/R (Fig. 8c). These results demonstrate that BT2 and Fer-1 significantly inhibited BCAA-induced neuronal ferroptosis following cerebral I/R injury.

We further monitored the CBF of each group 24 h after cerebral I/R, and the results showed that BT2 and Fer-1 obviously alleviated the CBF reduction compared with the VEH group (Fig. 8d). Furthermore, in the VEH group, Nissl staining displayed few Nissl bodies scattered throughout the ischemic area and an apparent decrease in the number of Nissl bodies. Surprisingly, BT2 and Fer-1 markedly ameliorated the reduction in Nissl staining and quantitative analysis showed that numbers of surviving cells in BT2 and Fer-1 groups were significantly increased compared with the VEH group (Fig. 8d). TTC staining showed that BT2 and Fer-1 could significantly decrease the infarct volume (%) induced by cerebral I/R injury (Fig. 8e). These results indicate that BT2 and Fer-1 protected against cerebral I/R-induced CBF reduction and neuronal damage during the first 24 h.

To further investigate the effect of BT2 and Fer-1 on functional recovery after cerebral I/R, several scorings were evaluated. We first observed the change of mortality within 14 days after cerebral I/R (Fig. 8g). The results show that 100% of sham group mice survived within 14 days, while only 66.7% of mice survived for 5 days after cerebral I/R, compared with 84.6% of mice in the BT2 group and 92.3% of mice in the Fer-1 group. At 14 days, survival rates dropped to 44.44%, 69.2%, and 61.5% in I/R, BT2, and Fer-1 groups, respectively. Accordingly, BT2 and Fer-1 could delay death after cerebral I/R. Longa scores indicated that significantly better neurological recovery in BT2 and Fer-1 groups compared with the VEH group at 5–7 days after cerebral I/R (Fig. 8h). In addition, the Garcia score, a more particular and comprehensive scale, further confirmed that mice in BT2 and Fer-1 groups had significantly better recovery in physical coordination, movement, and sensation from 3–14 days after cerebral I/R (Fig. 8h). We also performed gait analysis with the CatWalk system after cerebral I/R to objectively evaluate the effect of BT2 and Fer-1 (Fig. 8i). Animals in BT2 and Fer-1 groups displayed significant increases in regularity index (Fig. 8j), kinetic parameters (body speed) (Fig. 8k), and paw pressure (including print area, max contact area, mean intensity, max contact mean intensity, and max intensity) (Fig. 8l, m and Fig. S4c–h), as well as significant decreases in temporal parameters (swing time) (Fig. 8n) of the left forepaw and left hindpaw compared with the VEH group. Taken together, these results confirm that BT2 and Fer-1 significantly ameliorated neurological functional defects and gait disturbance after cerebral I/R.

## Discussion

Although previous studies demonstrated the significance of ferroptosis in ischemic disease, the underlying mechanisms remain unknown [8, 28]. Our metabolomics results suggest that PUFA was enriched as a substrate of the ferroptosis pathway. Brain tissue has high oxygen uptake and is rich in PUFA, which is a target of ROS and prone to lipid peroxidation [34]. In addition, compared with glial cells, neurons have lower levels of GSH to protect against ROS [35]. Thus, exploring the mechanism of neuronal loss is pivotal to understanding cerebral I/R injury. Here, we first demonstrated that BCAA catabolic gene expression was suppressed in neurons after cerebral I/R. Specifically, a decrease in PPM1K expression led to increased BCKDHA S293 phosphorylation, resulting in BCAA accumulation. Elevated BCAA played an important role in neuronal lipid ROS and ferroptosis, and may contribute to the progression of cerebral I/R injury.

A growing body of studies have implicated abnormal BCAA metabolism in I/R injury, multiple metabolic diseases, and tumors [30, 36–39]. However, current understanding regarding BCAA metabolic regulation under cerebral ischemic conditions is very limited. Our findings show that BCAA levels in serum and brain tissue were significantly increased 24 h after cerebral I/R. Maple syrup urine disease (MSUD), an inherited disease caused by mutations in the mitochondrial BCKDH complex, leads to the toxic accumulation of BCAA and its related metabolites, resulting in lethal and acute neurological deterioration [40]. The accumulation of BCAA and/or BCKA in MSUD caused oxidative stress and lipid peroxidation [13, 41].

In the heart, abnormal BCAA catabolism resulted in elevated circulating and tissue BCAA levels, which directly inhibited PDH activity and glucose metabolism, causing a shift from glucose oxidative metabolism to FAO [42]. In vitro experiments suggest that BCAA increased expression of neuronal FAO-related genes and weakened the antioxidant system capacity; furthermore, lipid peroxidation (MDA and 4-HNE) was increased. In vivo, FAO and lipid peroxidation were increased 24 h after cerebral I/R. A study of diabetic mouse models showed that BCAA administration can increase levels of oxidative stress (8-hydroxydeoxyguanosine and 4-HNE), while reducing antioxidants (GSH/GSSG) and superoxide dismutase (SOD) [39]. Thus, there are reasons to believe that BCAA is involved in the redox metabolic disorder observed after cerebral I/R. Because lipid peroxidation is closely related to ferroptosis, we speculated that ferroptosis after cerebral I/R was partially mediated by elevated BCAA. Our results suggest that BCAA induced significant changes in neuronal ferroptosis-related genes and proteins, and increased lipid ROS levels, confirming that BCAA can induce neuronal ferroptosis in vitro.

Our findings reveal that PPM1K expression was reduced in neuronal cells following cerebral I/R injury. Because PPM1K is reportedly highly expressed in the brain, mainly in neurons [43], its decrease may cause the failure or death of large numbers of neurons. Notably, BCKDHA activity was determined by assessing PPM1K and BCKDK. The cell type-specific expression of BCKDK has been confirmed in neurons [44], BCKDHA is reportedly located in both neurons and endothelial cells [45]. Thus, we also verified that PPM1K and BCKDHA expression mainly occurred in neurons. Our results show that mRNA and protein expression of BCKDK were not significantly altered after cerebral I/R. Therefore, only the decrease of PPM1K protein led to the decrease of BCKDHA activity, and disrupted BCAA oxidative metabolism ultimately led to BCAA accumulation. Furthermore, our results demonstrate that knockdown of PPM1K expression in neurons in vitro can induce increased level of ferroptosis in neurons. Previous studies reported that high levels of BCAA inhibit glucose metabolism, which in turn increase glucose uptake to repress cardiomyocyte BCAA oxidation metabolism genes and increase BCAA concentrations [28]. After cerebral I/R, uptake of glucose by brain cells increases [46], which may be an indirect mechanism of disrupted BCAA metabolism after cerebral I/R.

To further verify that elevated BCAA mediated ferroptosis after cerebral I/R, we enhanced BCAA metabolism pharmacologically to observe neurological recovery and changes in ferroptosis. Our results show that BT2 or Fer-1 intervention after cerebral I/R markedly reduced cerebral infarct volumes and neuronal death, and improved CBF and neurological recovery after cerebral I/R. BT2 inhibition of BCKDK activity can effectively activate BCKDH and significantly reduce p-BCKDHA levels [47]. Consistent with our findings, BCAA levels and neuronal p-BCKDHA S293 were significantly decreased by BT2. *Ppm1k*-KO mice display deficient BCAA catabolism that promotes heart failure and is associated with induced oxidative stress and metabolic disturbances; however, BT2 significantly mitigated cardiac dysfunction in these mice [38]. Gabapentin, a leucine analog and specific inhibitor of BCAT1 [48], reduced BCAA and MDA levels to ameliorate the decrease of GSH and increase of MDA in diabetic retinopathy [49]. In the present study, BT2 ameliorated BCAA-induced neuronal lipid peroxidation and ferroptosis.

## Conclusions

In conclusion, our findings show that BCAA mediated neuronal ferroptosis in cerebral I/R injury by regulating BCKDHA Ser293 phosphorylation, providing new understanding of the biochemistry driving ferroptosis in ischemic stroke. Moreover, pharmacological intervention of BCAA metabolism to inhibit ferroptosis may be a novel strategy for treatment of ischemic stroke.

### Supplementary information

Supplementary Material

Figure S1

Figure S2

Figure S3

Figure S4

Original Data File

A reproducibility checklist

## Data Availability

Data will be made available on request.
