# Supplementary material for: PPM1K mediates metabolic disorder of branched-chain amino acid and regulates cerebral ischemia-reperfusion injury by activating ferroptosis in neurons
Source: Cell Death Dis. 2023 Sep 26;14(9):634. doi: 10.1038/s41419-023-06135-x (PMC10522625; doi:10.1038/s41419-023-06135-x)
Supplement: Supplementary file 1 — Supplementary Material [file 41419_2023_6135_MOESM1_ESM.docx]

**Supplementary Materials**

**PPM1K mediates metabolic disorder of branched-chain amino acid and regulates cerebral ischemia-reperfusion injury by activating ferroptosis in neurons**

Tao Li^1, †^; Lili Zhao^1, †^; Ye Li ^1, †^; Meijuan Dang^1^; Jialiang Lu ^1^; Ziwei Lu ^1^; Qiao Huang^2^; Yang Yang^1^; Yuxuan Feng^1^; Xiaoya Wang^1^; Yating Jian^1^; Heying Wang^1^; Yingying Guo^3^; Lei Zhang^1^; Yu Jiang^1^; Songhua Fan^1^; Shengxi Wu^2^; Hong Fan ^1,^ *; Fang Kuang^2,^ *; Guilian Zhang ^1,^ *

^1^ Department of Neurology, the Second Affiliated Hospital of Xi'an Jiaotong University, Xi'an, 710004, Shaanxi, China

^2^ Department of Neurobiology, School of Basic Medicine, Fourth Military Medical University, 710032, Shaanxi, China.

^3^ Department of Pediatrics, the Second Affiliated Hospital of Xi'an Jiaotong University, Xi'an, 710004, Shaanxi, China

*Corresponding authors:

Hong Fan: fanhong_2005@126.com

Fang Kuang: kuangf@fmmu.edu.cn

Guilian Zhang:[zhgl_2006@xjtu.edu.cn](mailto:zhgl_2006@xjtu.edu.cn)

**Materials and methods**

**Data processing for metabolomics**

MS raw data (.wiff) files were converted to the mzXML format by ProteoWizard, and processed by R package XCMS (version 3.2) or ChromaTOF (V 4.3x; LECO, St. Joseph, MI, USA) software [1]. Processing included peak deconvolution, alignment, and integration. Minfrac and cut-off were set as 0.5 and 0.3, respectively. Peaks detected in less than half of QC samples or RSD>30% in QC samples were removed [2]. An in-house MS2 database was applied for metabolite identification.

**Measurement of BCAA concentration**

After reperfusion, brain tissues and serum were immediately collected for detection of BCAA or storage at −80°C. Tissues were homogenized according to the manufacturer’s instructions and the supernatant was analyzed with a commercial kit (K564-100, Biovision, Milpitas, CA, USA) to determine relative levels of BCAA. The absorbance of each sample was measured at 450 nm with a multimode microplate reader (Infinite M200, Tecan, Männedorf, Switzerland) and calculated based on the standard curve.

**PI/Hoechst staining**

Cells were grown in confocal dishes and treated with different reagents as described above. After washing cells with PBS, Hoechst 33342 and PI were added to the culture medium at final concentrations of 2 μg/mL and 5 μg/mL, respectively, and cells were incubated at 37°C for 15 min. Next, cells were washed and immediately observed and imaged with a confocal microscope (Olympus FV 3000). Finally, the rate of cell death was calculated by determining the PI^+^/ Hoechst^+^ ratio

**Culture of SH-SY5Y cells**

The SH-SY5Y cell line was obtained from American Type Culture Collection (Manassas, VA, USA) and cultured in Minimum Essential Media with F12 supplement and 10% fetal bovine serum at 37°C with 5% CO_2_. Cells were authenticated by short tandem repeat profiling and tested for mycoplasma contamination.

**Measurement of 4-HNE concentration**

Cells were seeded onto six-well plates at a density of 5 × 10^5^ cells/well and then harvested at the indicated assay end points. The concentration of 4-HNE in each sample was determined with a commercially available 4-HNE detection kit (ab238538, Abcam) according to the manufacturer’s protocol and calculated based on a standard curve.

**Measurement of Intracellular Fe^2+^ detection**

To detect intracellular Fe^2+^, FerroOrange fluorescent probe was used according to the manufacturer’s protocol (F374, Dojindo, Kumamoto, Japan). Cells were grown in confocal dishes and treated with medium or BCAA. After washing cells with PBS, 1 µM FerroOrange was added to the culture medium and cells were incubated at 37°C for 30 min. Next, cells were washed, immediately observed, and imaged with a confocal microscope (Olympus FV 3000). Finally, Fe^2+^ mean fluorescence intensity (MFI) was quantified with Image J software (National Institutes of Health, Bethesda, MD, USA).

**Transmission electron microscopy**

Ultrastructural changes of neurons after BCAA exposure were assessed with transmission electron microscopy. Cells were fixed in 3% glutaraldehyde overnight at 4°C. The next day, cells were immersed in 1% osmic acid for 30 min and then dehydrated in a graded series of ethanol (50%, 70%, 80%, 90%, 95%, and 100%). After staining samples with uranyl acetate and lead citrate, images were taken using an electron microscope (JEM-1230; JEOL, Tokyo, Japan).

**Measurement of lipid peroxidation with imaging flow cytometry**

SH-SY5Y cells were seeded in six-well plates (1 × 10^5^ per well) and then treated with BCAA or RSL3. Following staining with 2 μM C11-BODIPY 581/591, cells were washed three times with PBS and analyzed on an Amnis ImageStreamX (Luminex, Austin, TX, USA) flow cytometer [3, 4]. Imaging flow cytometry with a 488-nm blue laser (200 mW) was used for detection of oxidized C11 (Ch02), while side scatter images (Ch01) were produced from a dedicated 785-nm laser (2 mW). The sample flow rate was set to medium with 40× magnification. Ch01 was used to find focused cells based on Area_M01 versus Aspect Ratio _M01 (i.e., size vs. circularity) to gate single cells. A region (R1) was created to gate for a single population. At least 1 × 10^4^ events were recorded in R1 for every sample. Collected data were analyzed using ImageStream Data Exploration and Analysis Software (Luminex). The results of flow cytometric analysis of oxidized C11-BODIPY 581/591-labeled (Ch02) cells are expressed in MFI.

**Nissl staining**

After washing sections twice with PBS, the slides were immersed in Nissl staining solution (C0117, Beyotime) for 30 min at 50°C. Next, sections were rinsed with a series of ethanol concentrations from 50% to 100% and immersed twice in xylene for 5 min. Subsequently, sections were mounted under coverslips in neutral gum and air dried. Histological changes were observed with a VS200 microscope (Olympus) to assess neuronal damage.

Table S1 RT-PCR primer sequences

| Gene | Forward Primer(5'to3') | Reverse Primer (5'to3') |
| --- | --- | --- |
| *Slc7a5* | AAGGCACCAATCTGGACGTG | GGTTCGTCAGCACATAGACCA |
| *Bcat1* | CTGCCTCTGTTTTGCACTACGC | TCCTCACAGCAGATCGGCACAT |
| *Bcat2* | CAAAGGTGGAGACCAGCAGGTA | TGGCGGATACACTCCAACAGCT |
| *Ppm1k* | GAGTTATGCCCACCTGTCTGCA | CTGTCTCCAACACTGGCTACCA |
| *Bckdk* | GCCACAATGGAGAGTCACCTAG | CCAGGTCCTTATGAGCGATTCC |
| *Bckdha* | ATGGCTATGCCATCTCCACACC | CAAACACATCGTTGCCGTCCAC |
| *Acaa2* | ACACCTGGTTCACGAGTTAAG | GTTCTGGATGATCAGGGAGATG |
| *Fabp3* | TAGCATGACCAAGCCGACCA | ACCAGTTTGCCTCCGTCCAG |
| *Cd36* | GGACATTGAGATTCTTTTCCTCTG | GCAAAGGCATTGGCTGGAAGAAC |
| *Acadm* | GCTACAAGGTCCTGAGAAGTG | CTCCGTCAACTCGAAGCTAAA |
| *Cpt1b* | TACACGCATCCCAGGCAAAG | CGAGCCCTCATAGAGCCAAAC |
| *Acsl1* | TTCGCAGTGGCATCGTCAG | TGTGATCATCAGCCGGACTTTC |
| *Acsl4* | CTCACCATTATATTGCTGCCTGT | TCTCTTTGCCATAGCGTTTTTCT |
| *Gpx4* | GATGGAGCCCATTCCTGAACC | CCCTGTACTTATCCAGGCAGA |
| xCT | CTGCTCGTAATACGCCCTGG | CCAGCTGACACTCGTGCTAT |
| *Gls2* | CGTCCGGTACTACCTCGGT | TGTCCCTCTGCAATAGTGTAGAA |
| *Lpcat3* | GACGGGGACATGGGAGAGA | GTAAAACAGAGCCAACGGGTAG |
| *Tfrc* | GTTTCTGCCAGCCCCTTATTAT | GCAAGGAAAGGATATGCAGCA |
| *Ptgs2* | TTCAACACACTCTATCACTGGC | AGAAGCGTTTGCGGTACTCAT |
| *Ncoa4* | GAACCATCAGGACACATGGAAA | AGGAGCCATAGCCTTGGGT |
| *Atp5g3* | TCTGCATCAGTGTTATCTCGGC | CACCAGAACCAGCAACTCCTA |
| *Aifm2* | GGCAAGTTTAACGAGGTGTCC | CCTTCTCAGGGTACTCGGTTTTA |
| β-actin | GGCTGTATTCCCCTCCATCG | CCAGTTGGTAACAATGCCATGT |

Table S2 Sequences of shRNA

| shRNA | Target Sequence (5'to3') |
| --- | --- |
| PPM1K shRNA | ACTTGCAATGACAAGGAGTAT |
| Scramble shRNA | CCTAAGGTTAAGTCGCCCTCG |

**Results:**

Table S3 Amino acids expression between MCAO group and sham group

|  | MCAO | Sham | FC | *P* |
| --- | --- | --- | --- | --- |
|  | Mean | Mean |  |  |
| Glutamine | 0.591 | 0.120 | 4.922 | 0.021 |
| Beta-glutamic acid | 0.909 | 0.194 | 4.684 | 0.015 |
| Valine | 9.943 | 3.465 | 2.869 | 0.016 |
| Isoleucine | 5.075 | 2.044 | 2.483 | 0.013 |
| Serine | 21.797 | 9.423 | 2.313 | 0.018 |
| Alanine | 50.873 | 25.290 | 2.012 | 0.022 |
| Proline | 8.417 | 4.280 | 1.967 | 0.016 |
| Acetylcysteine | 0.0006 | 0.0004 | 1.644 | 0.041 |

FC：Fold change; MCAO: middle cerebral artery occlusion.

Table S4 Fatty acids expression between MCAO group and sham group (Top 10)

|  | MCAO | Sham | FC | *P* |
| --- | --- | --- | --- | --- |
|  | Mean | Mean |  |  |
| Nervonic acid | 0.033 | 0.139 | 0.237 | 0.000* |
| Arachidonic acid | 3.640 | 13.853 | 0.263 | 0.000* |
| 16b-hydroxyestradiol | 0.003 | 0.010 | 0.334 | 0.000* |
| Docosatrienoic acid | 0.018 | 0.053 | 0.335 | 0.002 |
| Oleic acid | 3.104 | 8.132 | 0.381 | 0.000* |
| Cis-9-palmitoleic acid | 0.240 | 0.575 | 0.417 | 0.000* |
| Tetracosanoic acid | 0.013 | 0.028 | 0.450 | 0.015 |
| Gamma-linolenic acid | 0.080 | 0.165 | 0.485 | 0.000* |
| Eicosadienoic acid | 0.053 | 0.106 | 0.497 | 0.001 |
| Arachidic acid | 0.017 | 0.028 | 0.617 | 0.019 |

FC：Fold change; MCAO: middle cerebral artery occlusion; *: *P* < 0.0001.


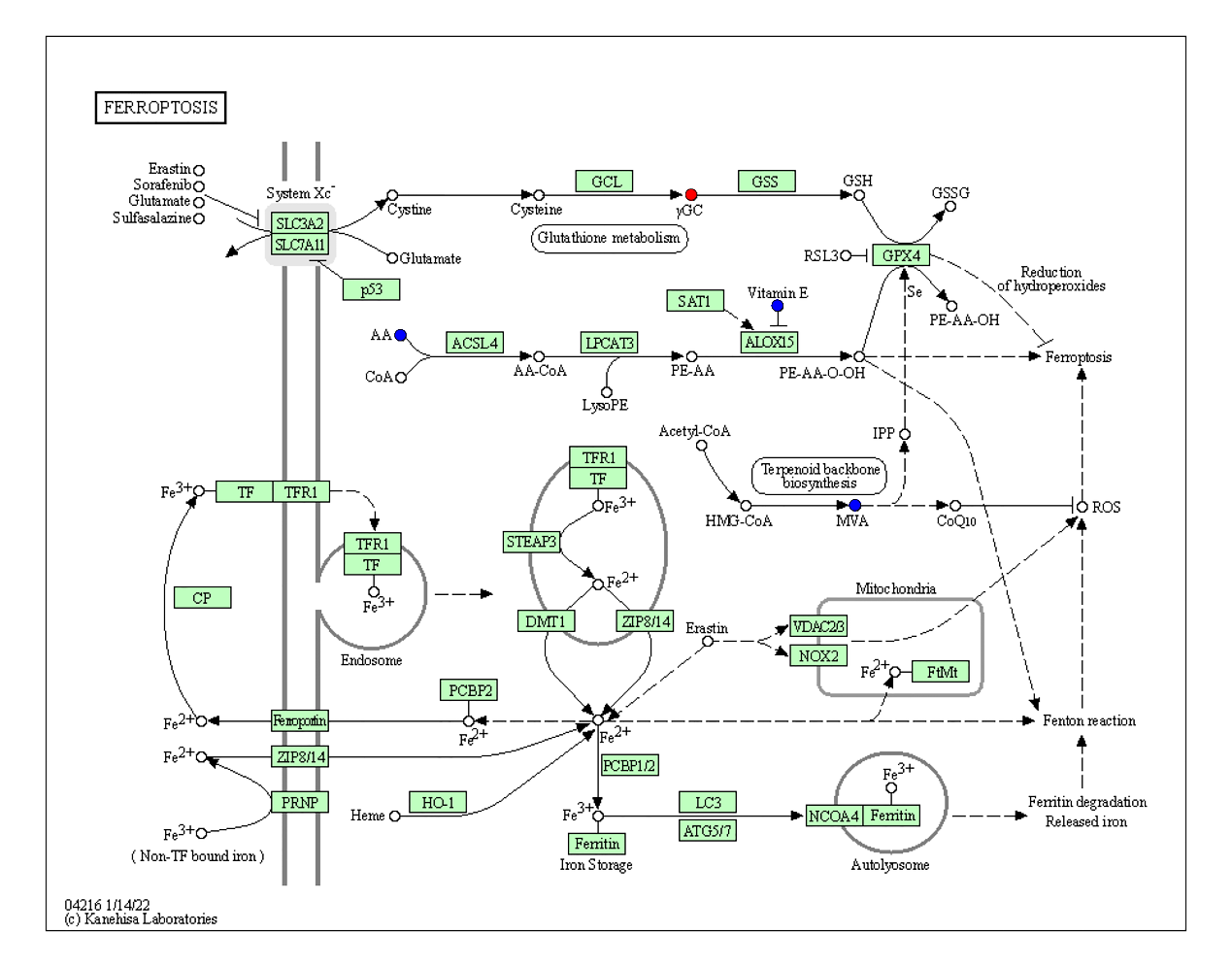


**Fig. S1** KEGG Metabolic pathways of ferroptosis with red/blue dots representing the differentially expressed compounds. blue: downregulated, red: upregulated


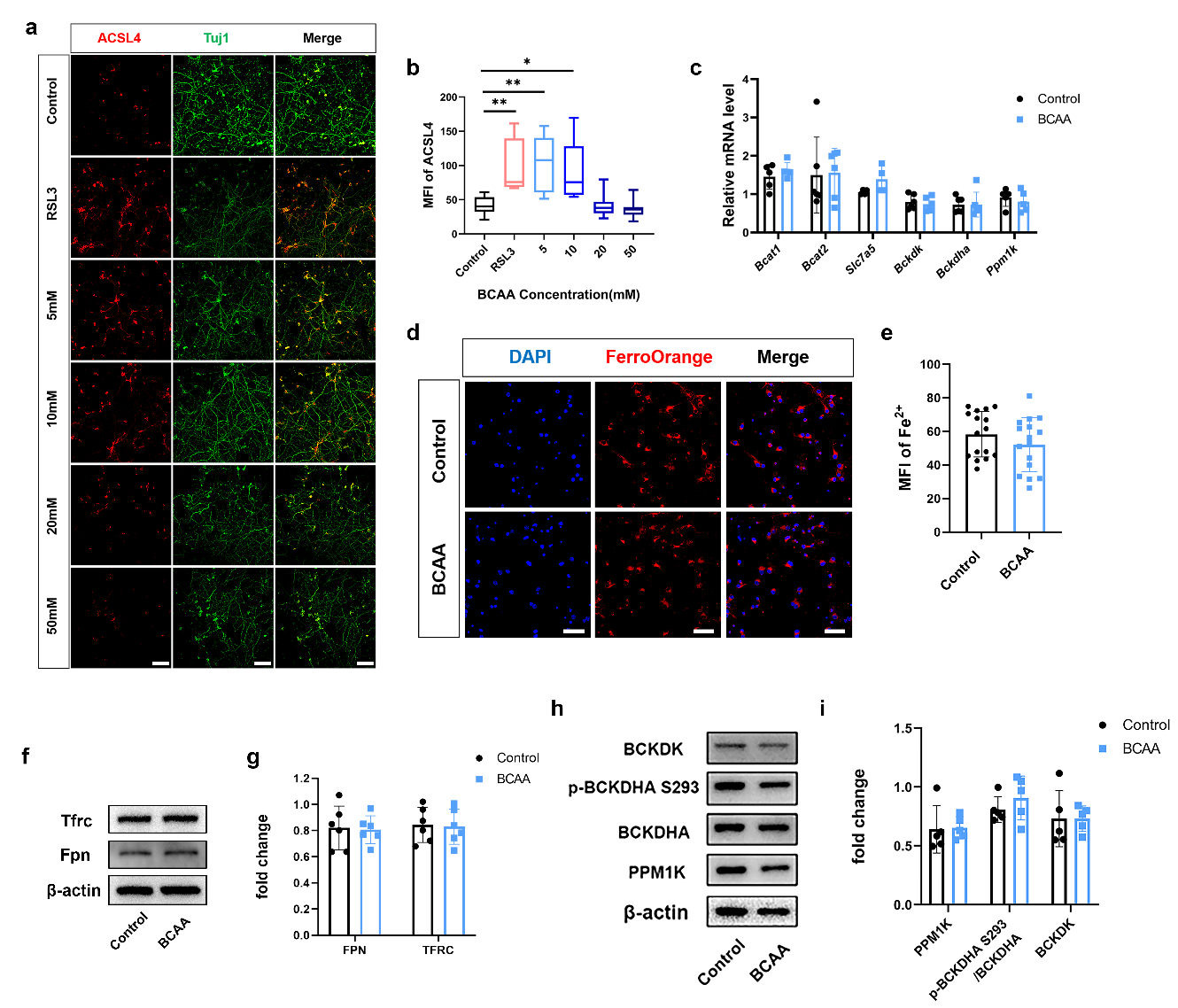


**Fig. S2** BCAA induced ACSL4 expression in neuron and had no effect on BCAA-related enzyme. (**a**): Representative images of ACSL4 and Tuj1 double-stained neurons treated with RSL3 (5 μM) for 2 h and 5, 10, 20, or 50 mM BCAA for 24 h. (**b**): Quantitative analysis mean immunofluorescence intensities (MFI) of ACSL4-positive cells in each group of neurons. (**c**): mRNA expression of BCAA-associated transcripts genes *Bcat1*, *Bcat2*, *Slc7a5*, *Bckdk*, *Bckdha* and *Ppm1k* in neurons treated with medium and BCAA (5 mM) after 24 h. (**d, e**): Representative images of Fe^2+^ and DAPI staining and quantification of Fe^2+^ MFI in two groups. (**f, g**): Representative immunoblot and statistical analysis of Fpn and Tfrc. (**h, i**): Representative immunoblot and statistical analysis of BCAA-related enzymes BCKDK, p-BCKDHA S293, BCKDHA and PPM1K from neurons under normal or BCAA (5 mM)-treated conditions, β-actin was used as a loading control. All experiments were performed at least three independent times. All data are presented as mean ± SD, **P*<0.05, ***P*<0.01


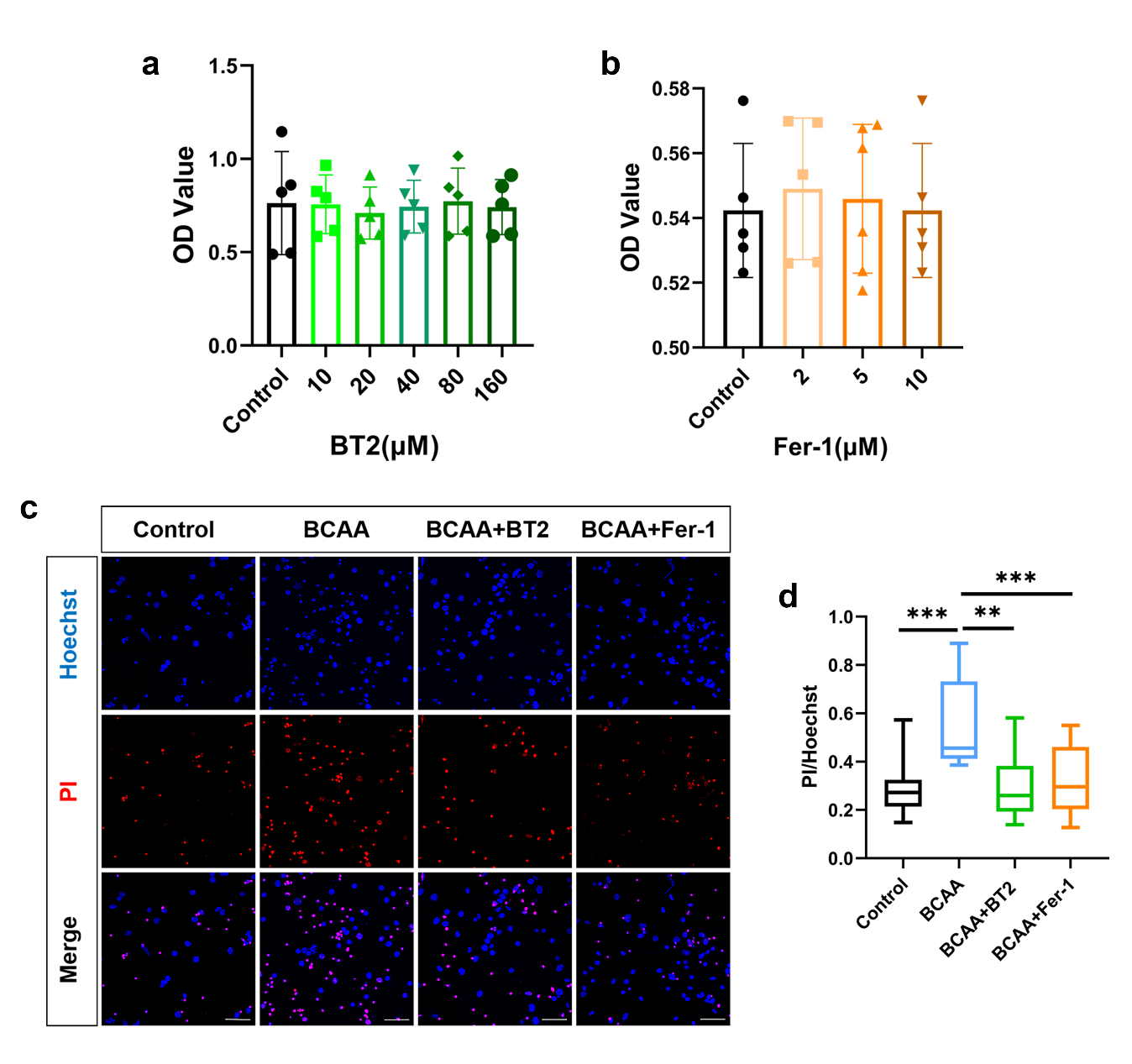


**Fig. S3** BT2 and Fer-1 mitigated BCAA-induced neuronal ferroptosis. (**a**): Cell viability of neurons was detected by CCK-8 assay following incubation with 10, 20, 40, 80 and 160 μM BT2 for 24h. (**b**): Cell viability of neurons was detected by CCK-8 assay following incubation with 2, 5 and 10 μM Fer-1 for 24h. (**c, d**): Neurons were pretreated with BT2 (80 μM) or Fer-1 (5 μM) for 4 h and then incubated with BCAA (5mM) for 24 h. (**c**): Representative images of PI-Hoechst double-stained neurons and (**d**) quantification of PI-positive/Hoechst-positive cells. Scale bar = 50μm. All experiments were performed at least three independent times. All data are presented as mean ± SD, ***P*<0.01, ****P*<0.001


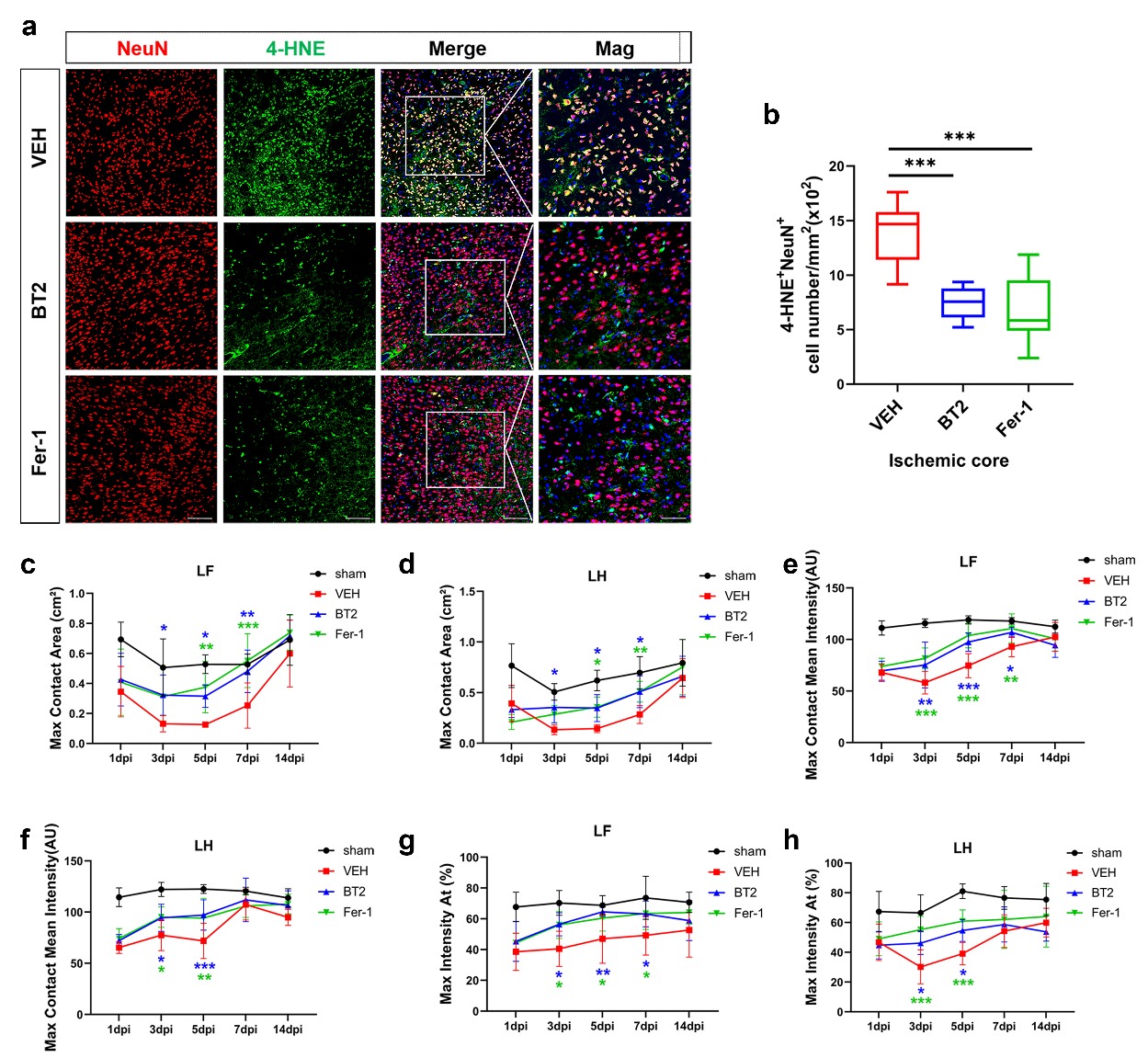


**Fig. S4** BT2 and Fer-1 alleviated ferroptosis and promoted motor functional recovery after cerebral I/R. **(a)** Representative images of NeuN/4-HNE staining in the ischemic core. Scale bar = 100 μm. Representative magnified images are shown on the far right. Scale bar = 50 μm. **(b)** Quantitative analysis of p-BCKDHA S293-positive and 4-HNE-positive cells in the ischemic core among VEH, BT2, and Fer-1 groups (n = 14–18images from four animals/group, one-way ANOVA followed by multiple comparisons). CatWalk analysis showed BT2 and Fer-1 significantly increased max contact area (**c, d**), max contact mean intensity (**e, f**), max intensity at (**g, h**) of the left forepaw and left hindpaw compared with the VEH group. n = 8–10/group, two-way ANOVA (Bonferroni’s multiple comparison test). LF: left forepaw, LH: left hindpaw. All data are presented as mean ± SD, **P*<0.05, ***P*<0.01, ****P*<0.001

**References:**

1. Kind T, Wohlgemuth G, Lee DY, Lu Y, Palazoglu M, Shahbaz S, et al. FiehnLib: mass spectral and retention index libraries for metabolomics based on quadrupole and time-of-flight gas chromatography/mass spectrometry. Anal Chem. 2009;81(24):10038-10048. <http://dx.doi.org/10.1021/ac9019522>.

2. Dunn WB, Broadhurst D, Begley P, Zelena E, Francis-McIntyre S, Anderson N, et al. Procedures for large-scale metabolic profiling of serum and plasma using gas chromatography and liquid chromatography coupled to mass spectrometry. Nat Protoc. 2011;6(7):1060-1083. <http://dx.doi.org/10.1038/nprot.2011.335>.

3. Fei C, Lillico DME, Hall B, Rieger AM, Stafford JL. Connected component masking accurately identifies the ratio of phagocytosed and cell-bound particles in individual cells by imaging flow cytometry. Cytometry A. 2017;91(4):372-381. <http://dx.doi.org/10.1002/cyto.a.23050>.

4. More TA, Dalal B, Devendra R, Warang P, Shankarkumar A, Kedar P. Applications of imaging flow cytometry in the diagnostic assessment of red cell membrane disorders. Cytometry B Clin Cytom. 2020;98(3):238-249. <http://dx.doi.org/10.1002/cyto.b.21857>.
