# Supplementary figures and images for: PPM1K mediates metabolic disorder of branched-chain amino acid and regulates cerebral ischemia-reperfusion injury by activating ferroptosis in neurons
Source: Cell Death Dis. 2023 Sep 26;14(9):634. doi: 10.1038/s41419-023-06135-x (PMC10522625; doi:10.1038/s41419-023-06135-x)

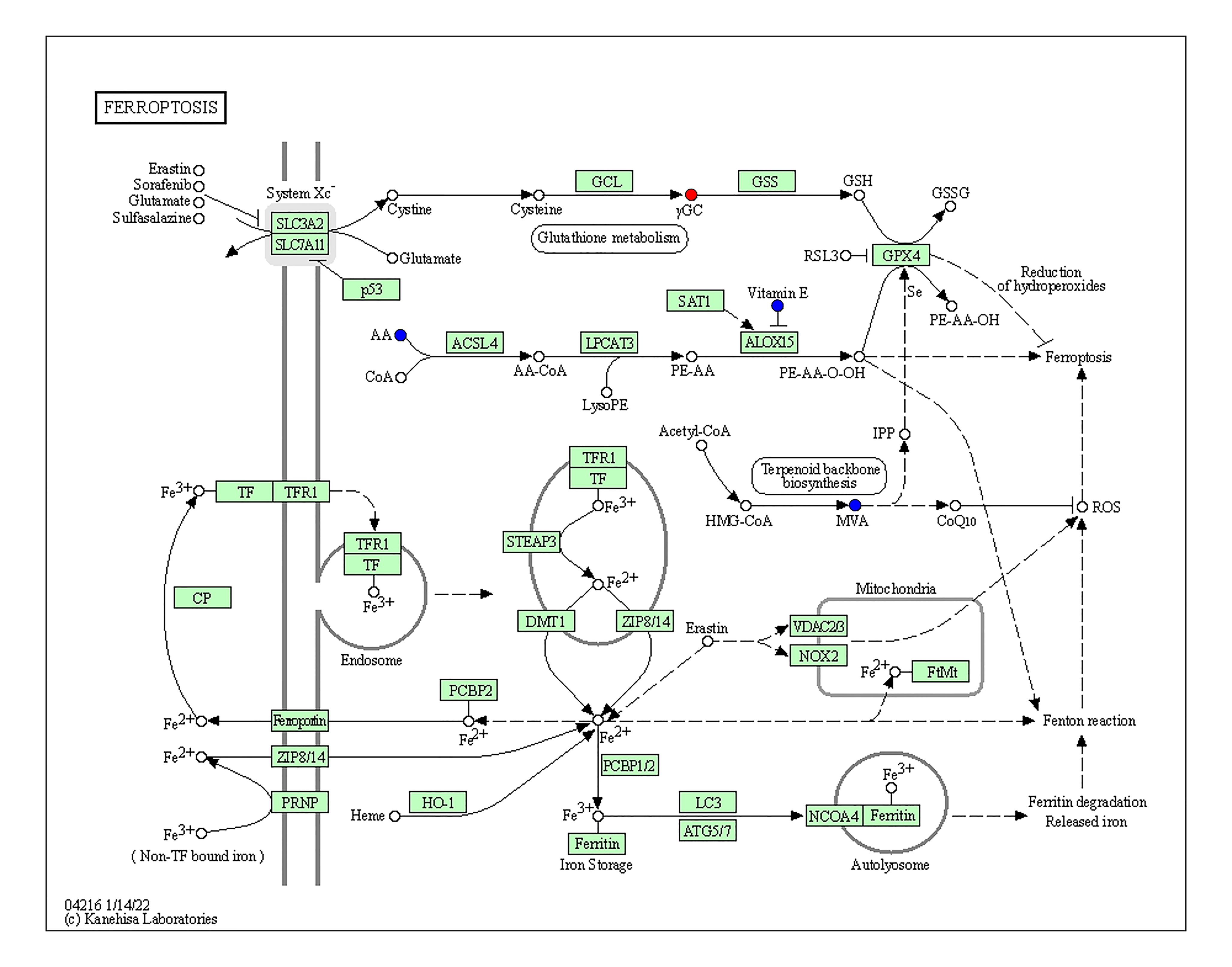

Supplement: Supplementary file 2 — Figure S1 [file 41419_2023_6135_MOESM2_ESM.tif]

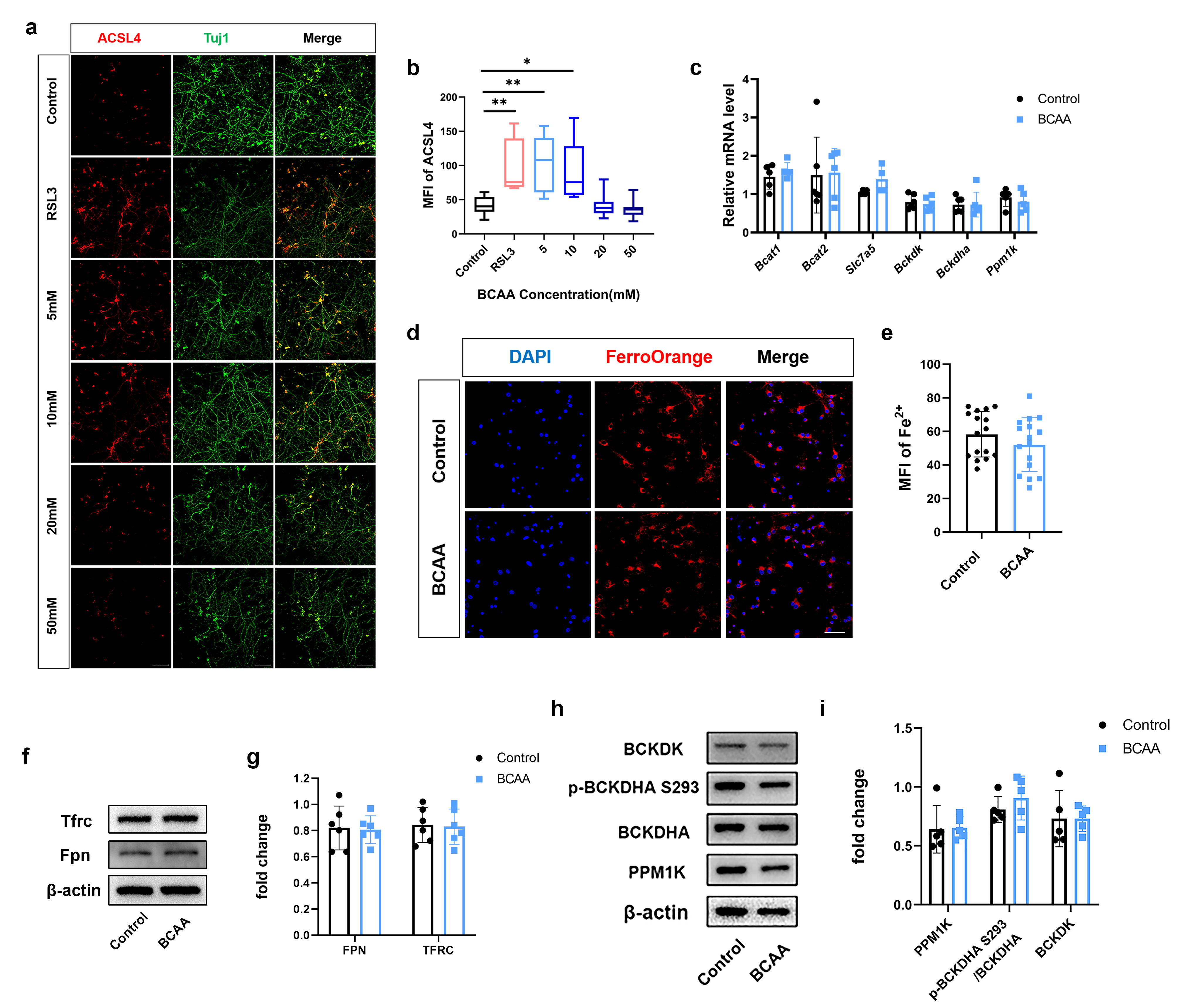

Supplement: Supplementary file 3 — Figure S2 [file 41419_2023_6135_MOESM3_ESM.tif]

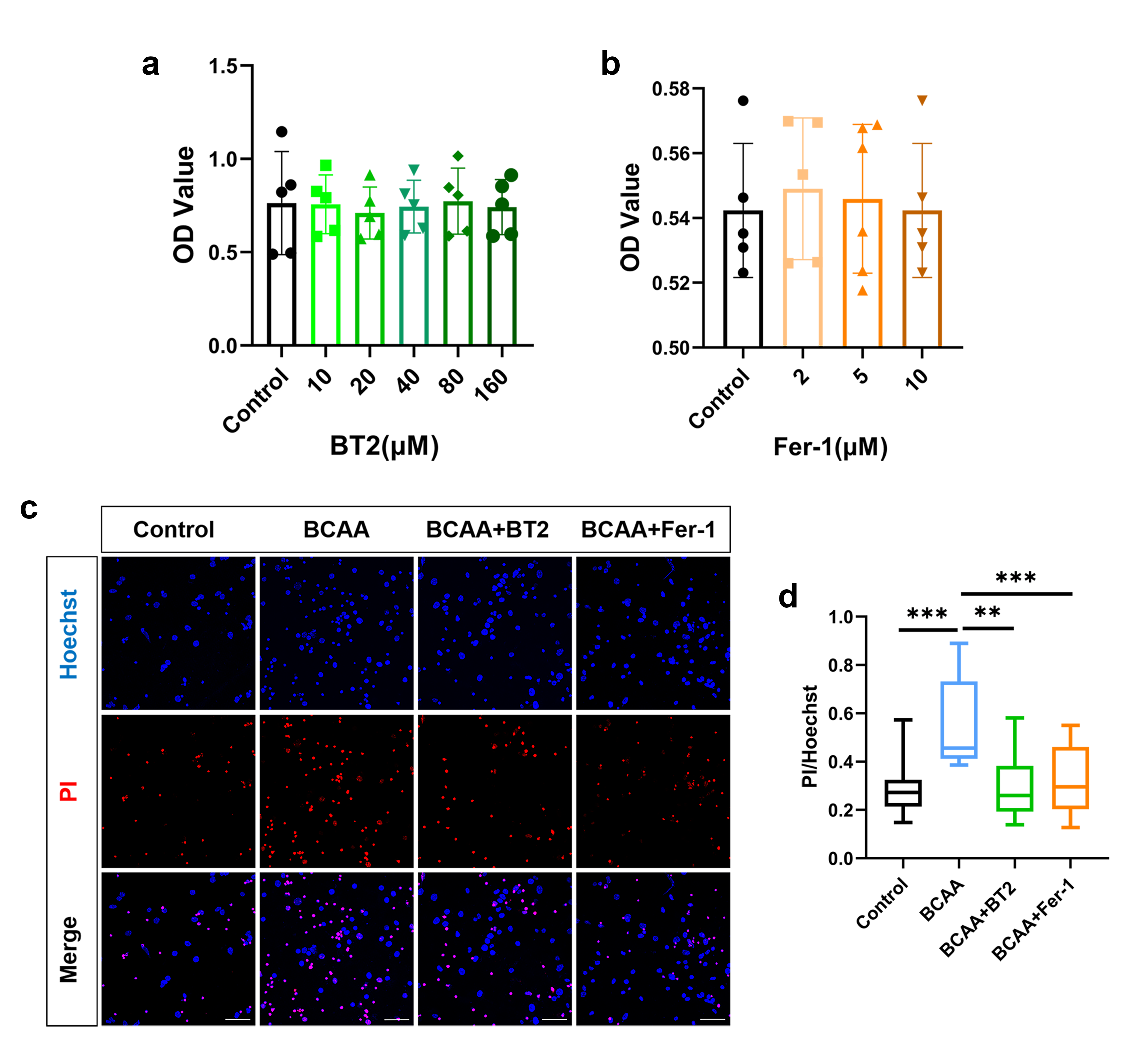

Supplement: Supplementary file 4 — Figure S3 [file 41419_2023_6135_MOESM4_ESM.tif]

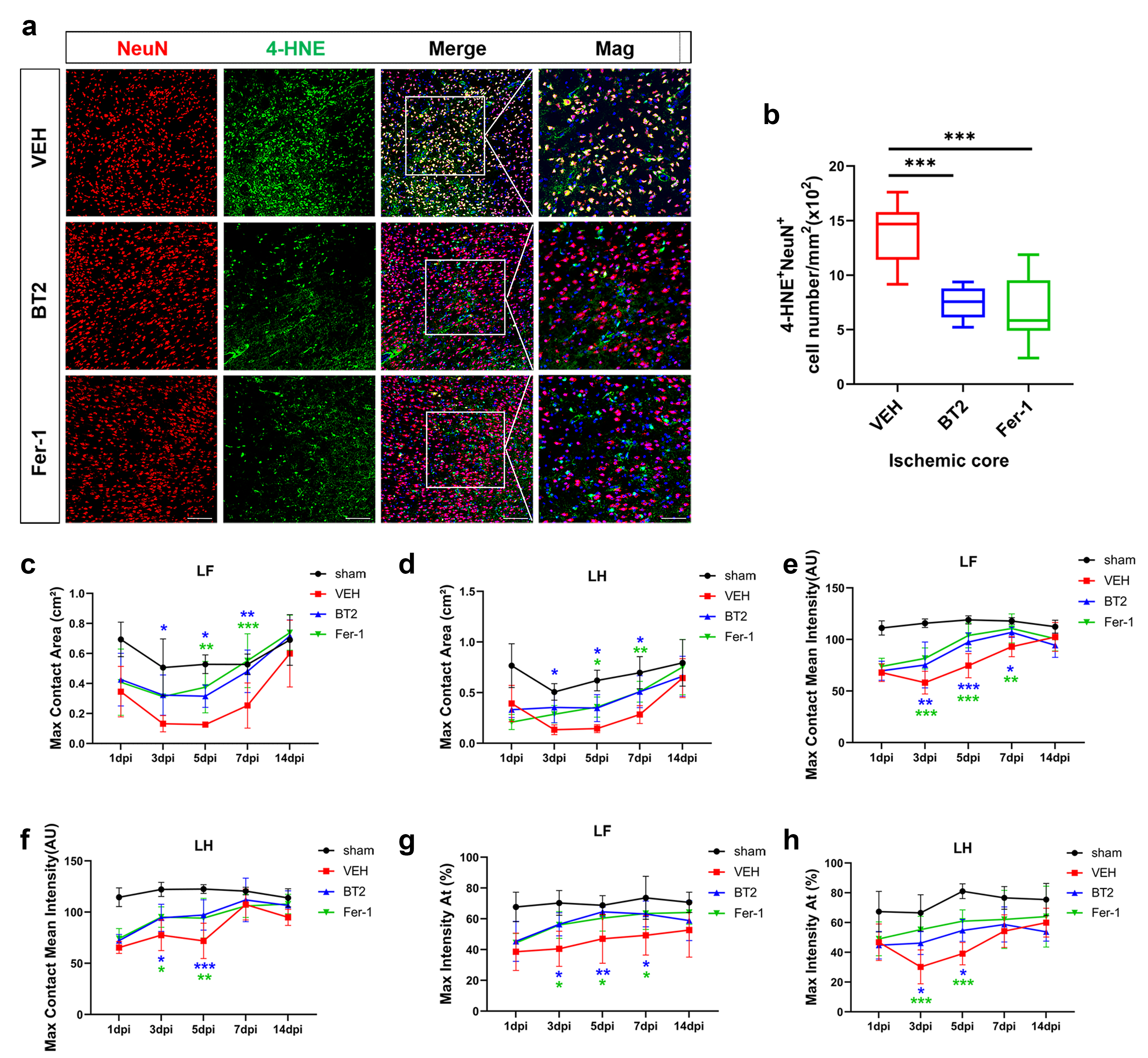

Supplement: Supplementary file 5 — Figure S4 [file 41419_2023_6135_MOESM5_ESM.tif]
