# Supplementary material for: PPM1K mediates metabolic disorder of branched-chain amino acid and regulates cerebral ischemia-reperfusion injury by activating ferroptosis in neurons
Source: Cell Death Dis. 2023 Sep 26;14(9):634. doi: 10.1038/s41419-023-06135-x (PMC10522625; doi:10.1038/s41419-023-06135-x)
Supplement: Supplementary file 6 — Original Data File [file 41419_2023_6135_MOESM6_ESM.docx]

**Supplementary Material**

**PPM1K mediates metabolic disorder of branched-chain amino acid and regulates cerebral ischemia-reperfusion injury by activating ferroptosis in neurons**

Tao Li^1, †^; Lili Zhao^1, †^; Ye Li ^1, †^; Meijuan Dang^1^; Jialiang Lu ^1^; Ziwei Lu ^1^; Qiao Huang^2^; Yang Yang^1^; Yuxuan Feng^1^; Xiaoya Wang^1^; Yating Jian^1^; Heying Wang^1^; Yingying Guo^3^; Lei Zhang^1^; Yu Jiang^1^; Songhua Fan^1^; Shengxi Wu^2^; Hong Fan ^1,^ *; Fang Kuang^2,^ *; Guilian Zhang ^1,^ *

^1^ Department of Neurology, the Second Affiliated Hospital of Xi'an Jiaotong University, Xi'an, 710004, Shaanxi, China

^2^ Department of Neurobiology, School of Basic Medicine, Fourth Military Medical University, 710032, Shaanxi, China.

^3^ Department of Pediatrics, the Second Affiliated Hospital of Xi'an Jiaotong University, Xi'an, 710004, Shaanxi, China

*Corresponding authors:

Hong Fan: fanhong_2005@126.com

Fang Kuang: kuangf@fmmu.edu.cn

Guilian Zhang: [zhgl_2006@xjtu.edu.cn](mailto:zhgl_2006@xjtu.edu.cn)

^†^ These authors have contributed equally to this work and share first authorship.


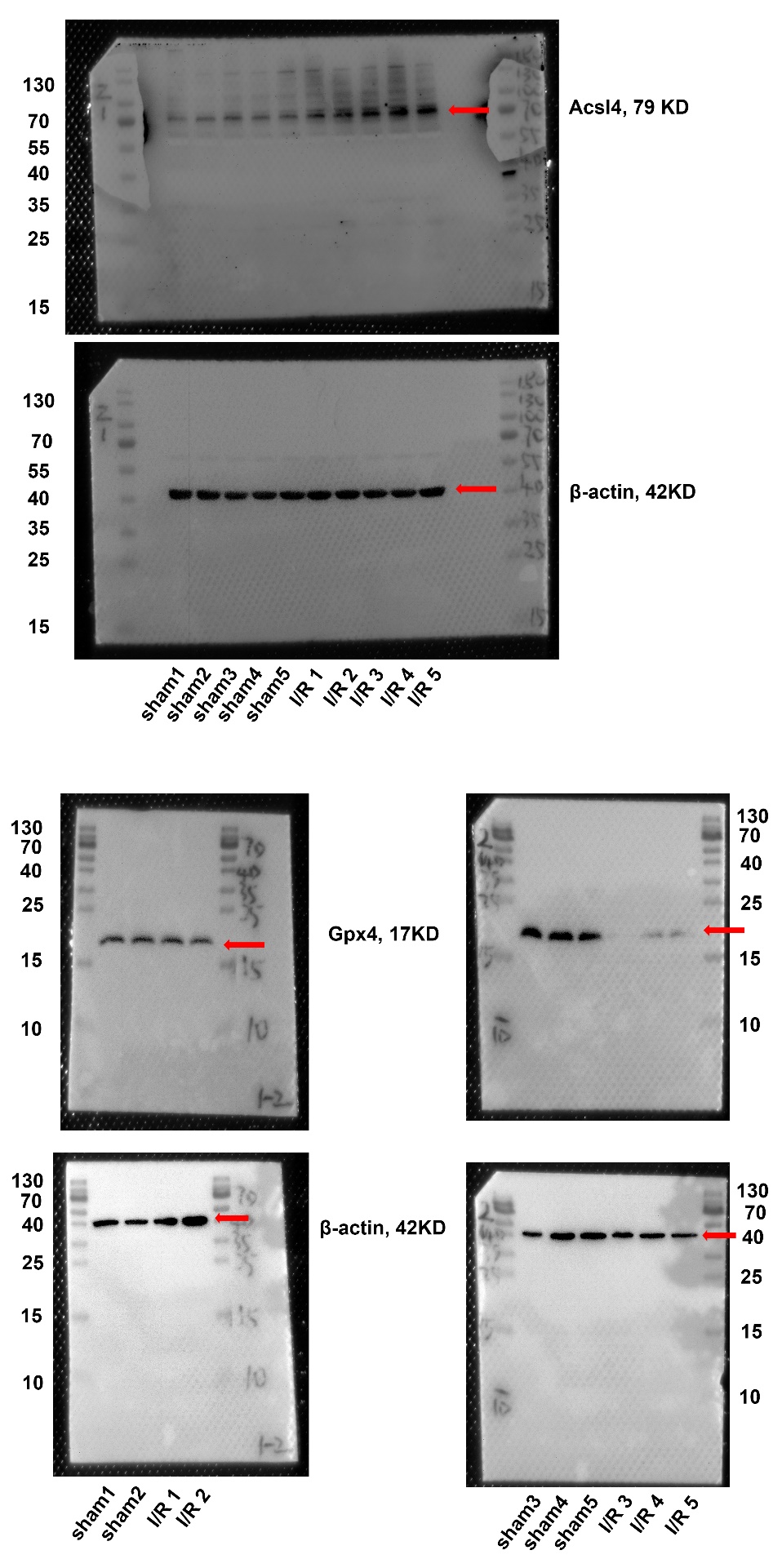


Figure. 1 The images of original, full-length Western blot gel of Fig. 2 (h, i). Red asterisk indicates the objective band.


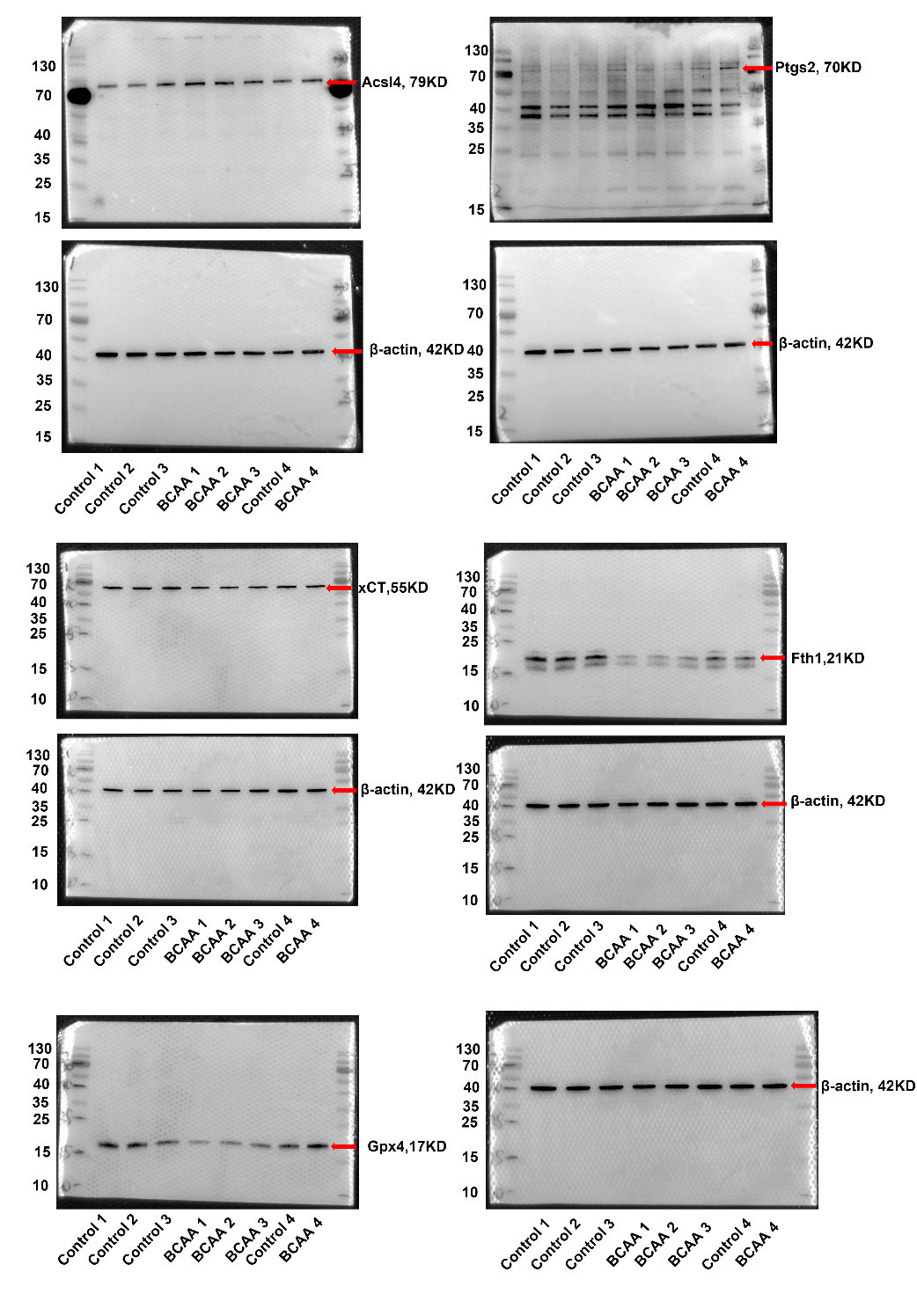


Figure. 2 The images of original, full-length Western blot gel of Fig. 4 (k, l). Red asterisk indicates the objective band.


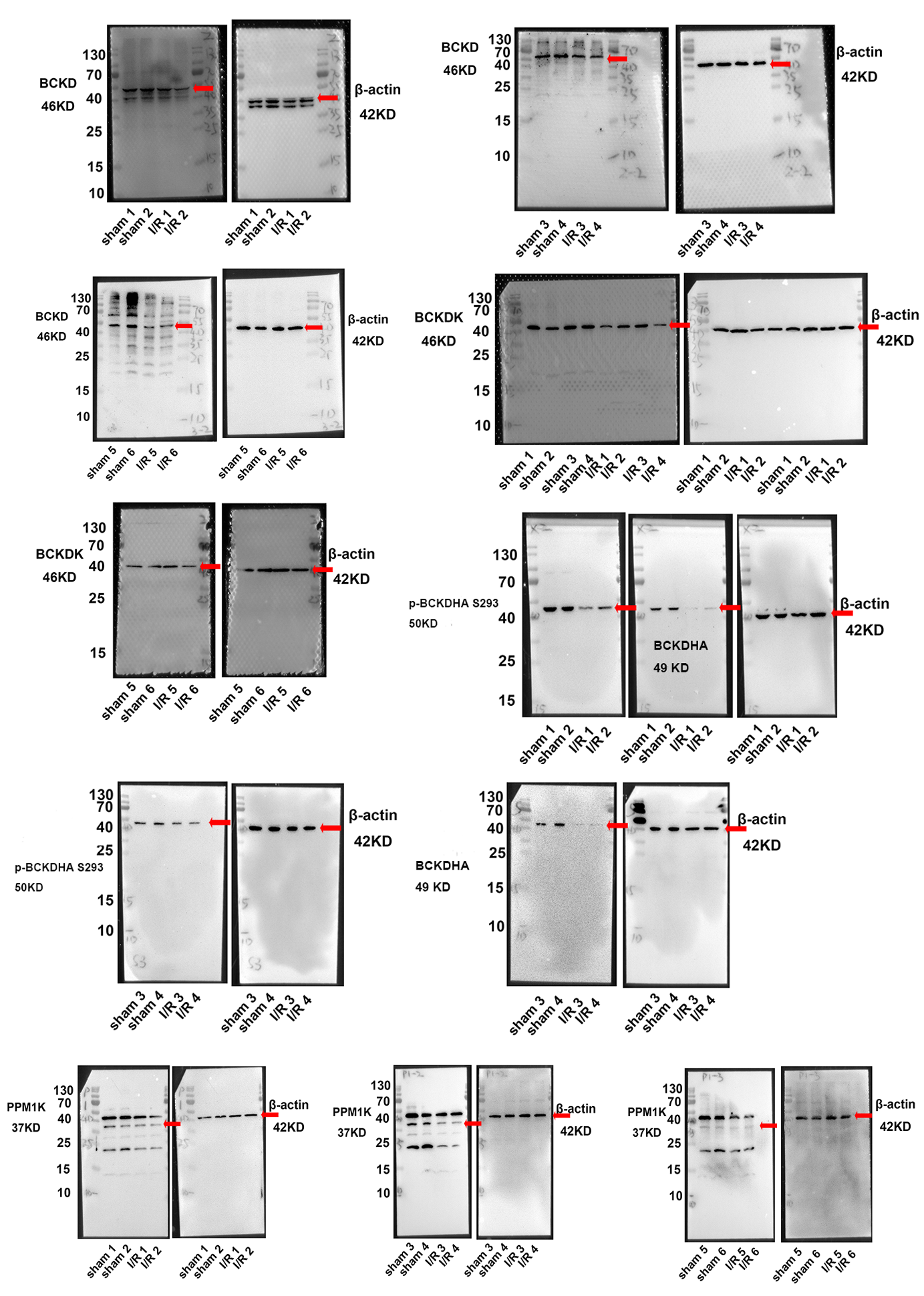


Figure. 3 The images of original, full-length Western blot gel of Fig. 6 (b, c). Red asterisk indicates the objective band.


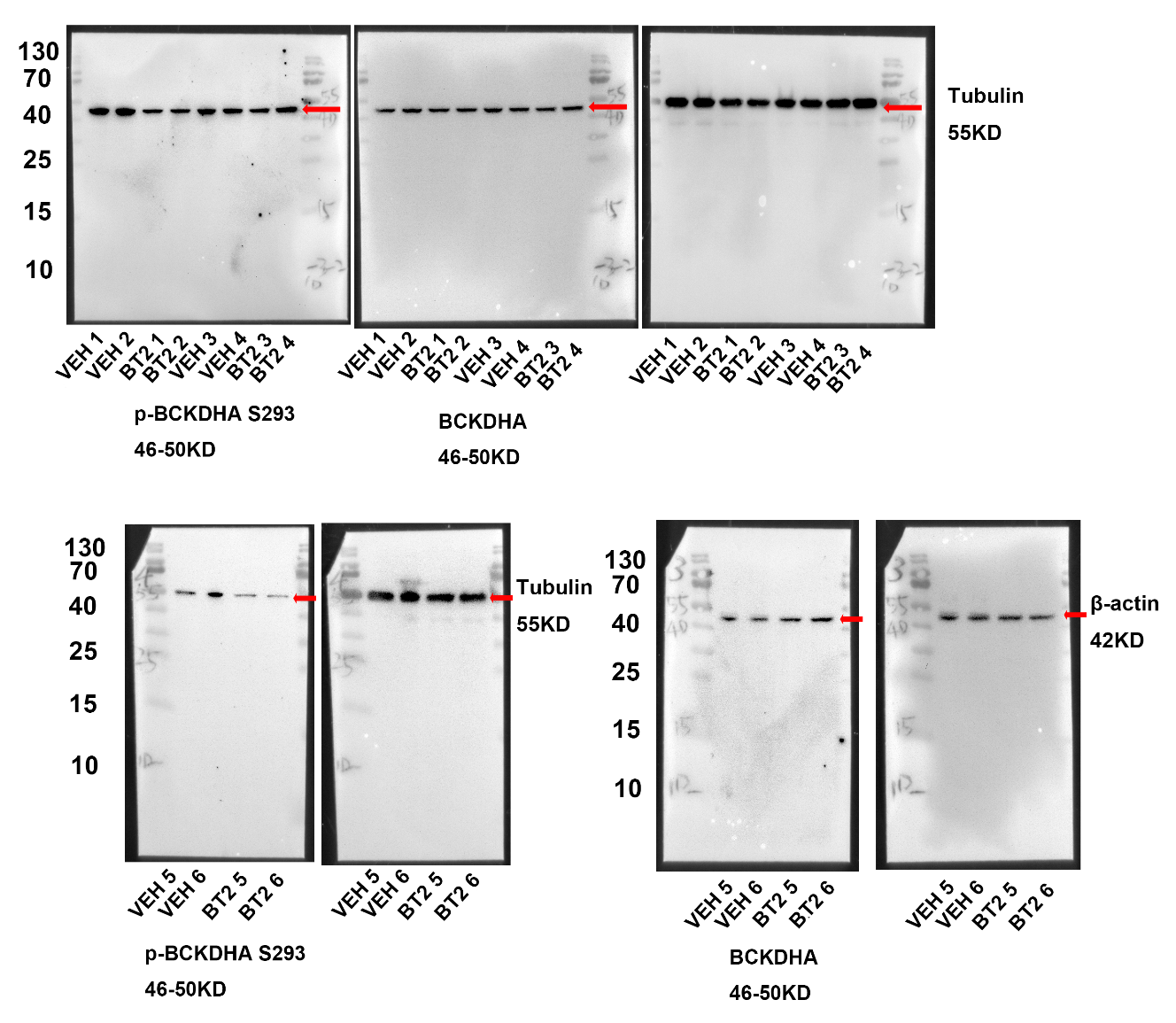
Figure. 4 The images of original, full-length Western blot gel of Fig. 6 (k, l). Red asterisk indicates the objective band.


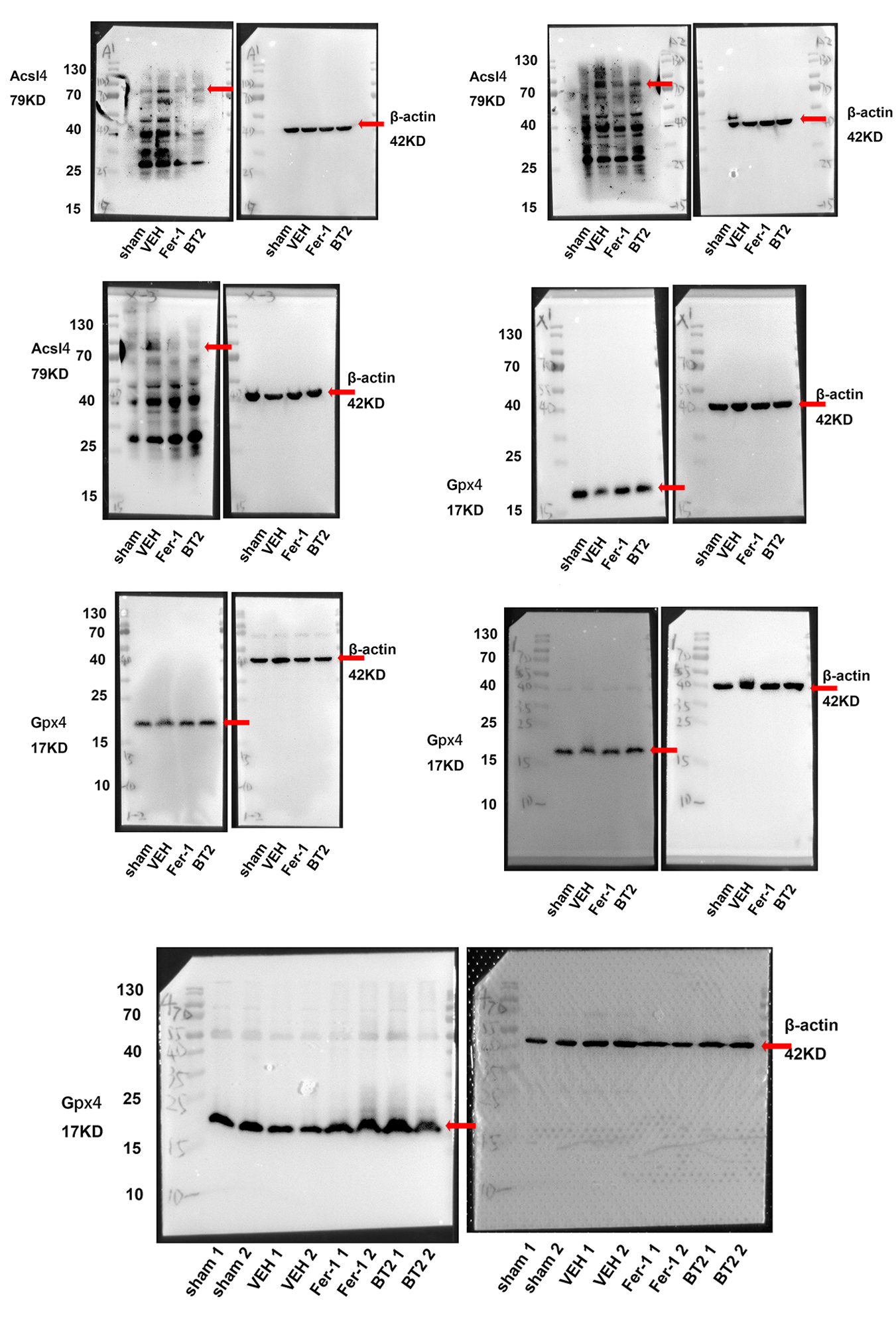


Figure. 5 The images of original, full-length Western blot gel of Fig. 7 (j, k). Red asterisk indicates the objective band.


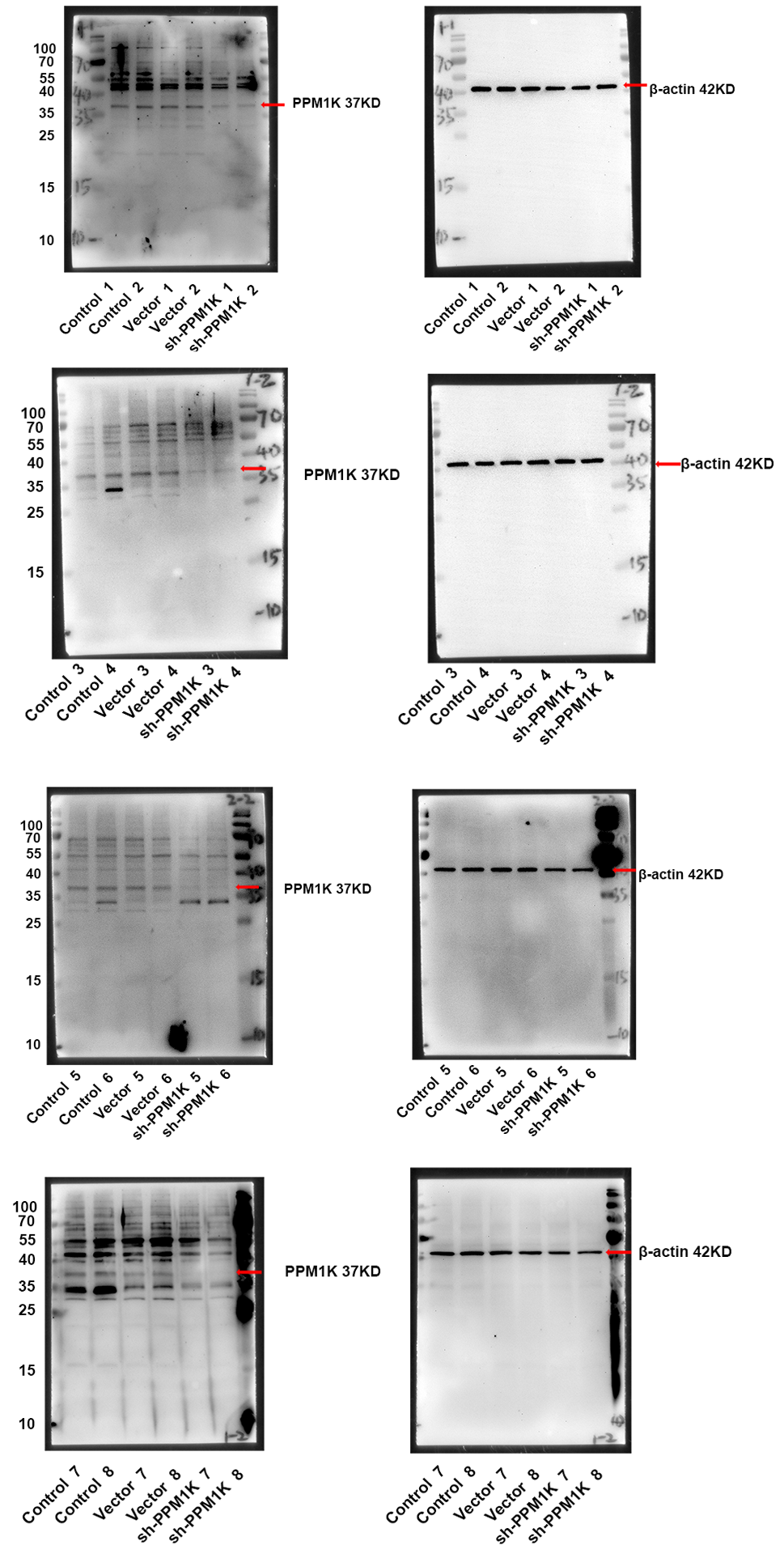


Figure. 6 The images of original, full-length Western blot gel of Fig. 7 (g). Red asterisk indicates the objective band.


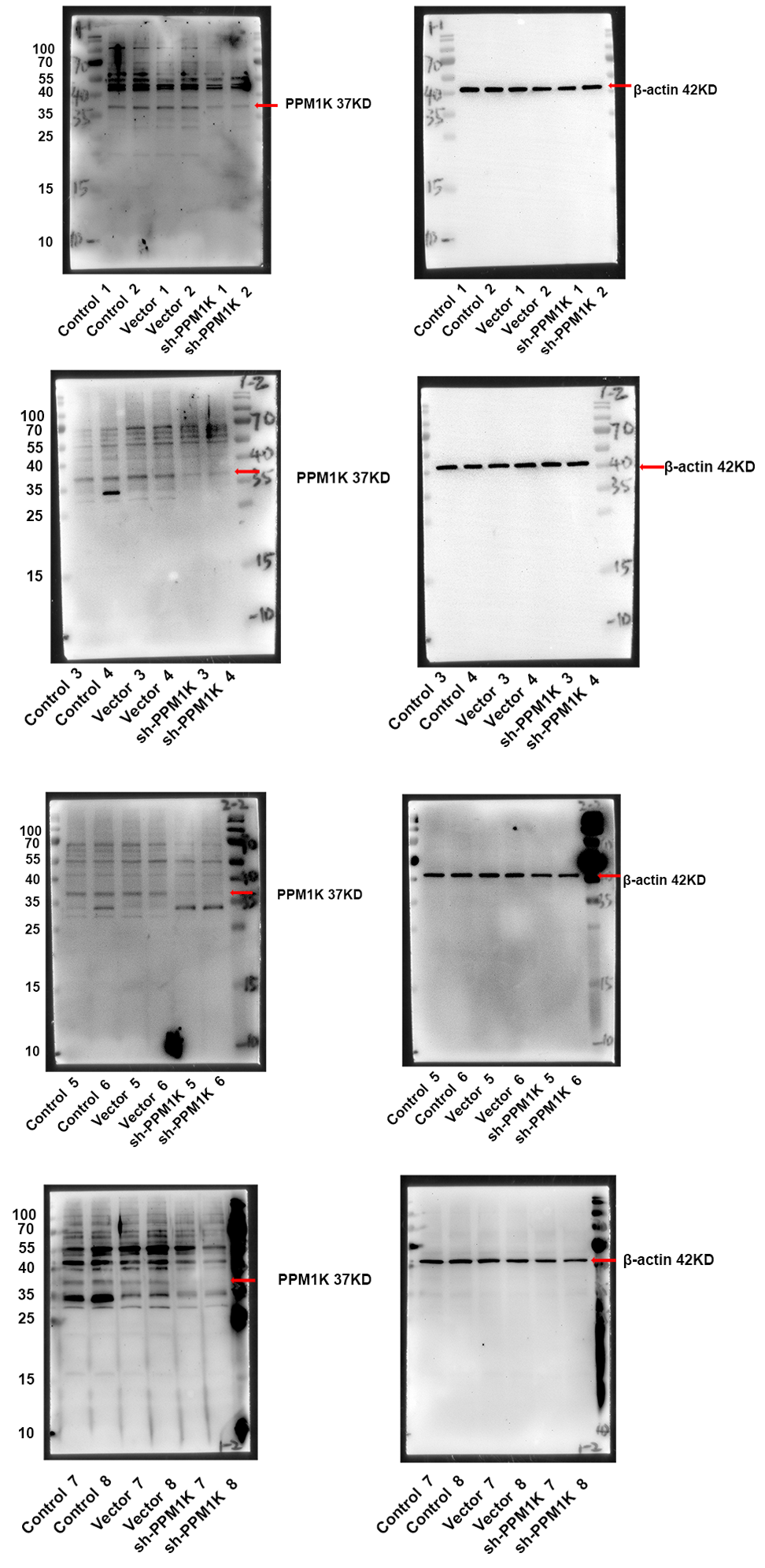


Figure. 7 The images of original, full-length Western blot gel of Fig. 7 (i). Red asterisk indicates the objective band.


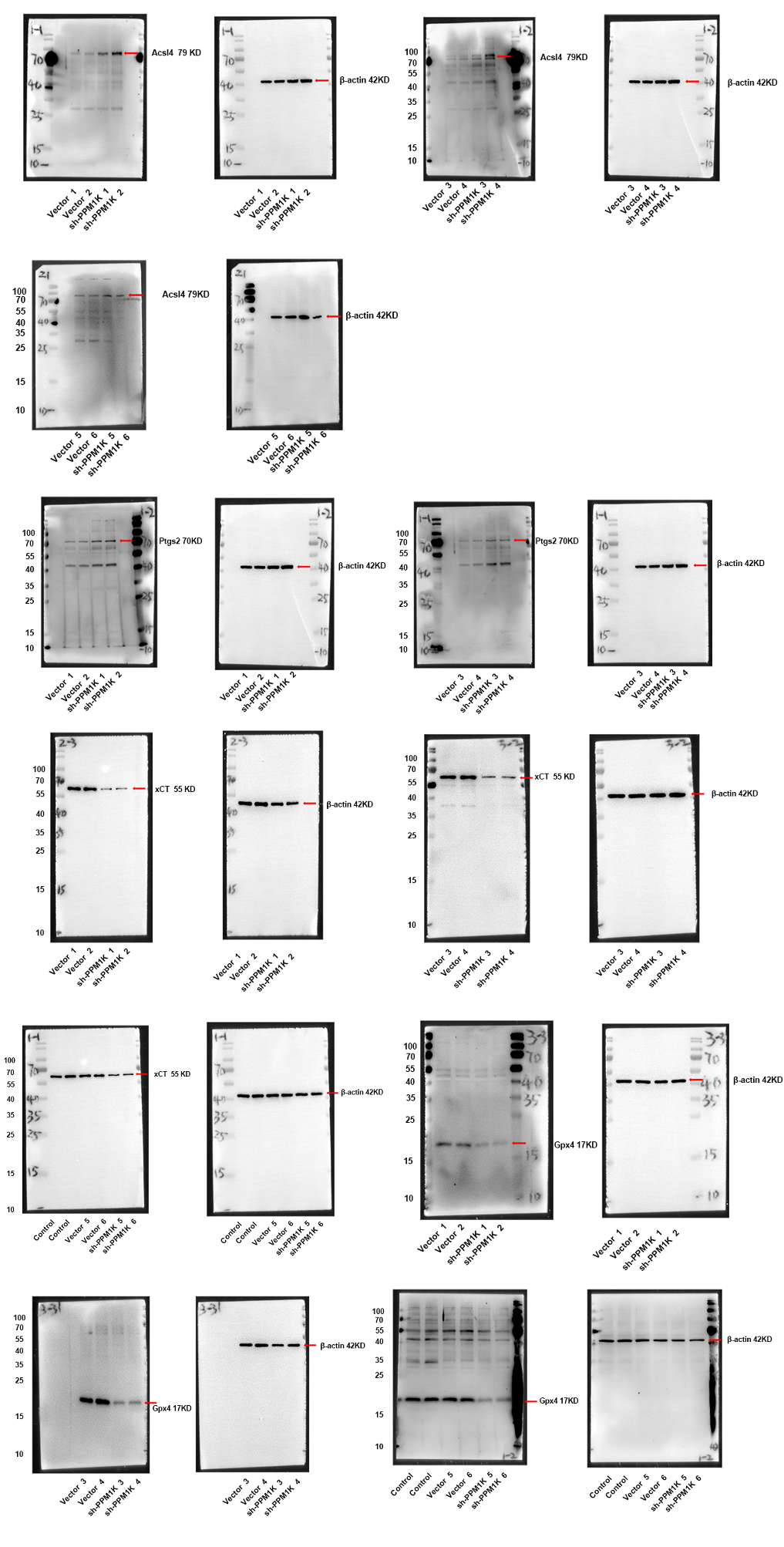


Figure. 8 The images of original, full-length Western blot gel of Fig. 7 (m). Red asterisk indicates the objective band.


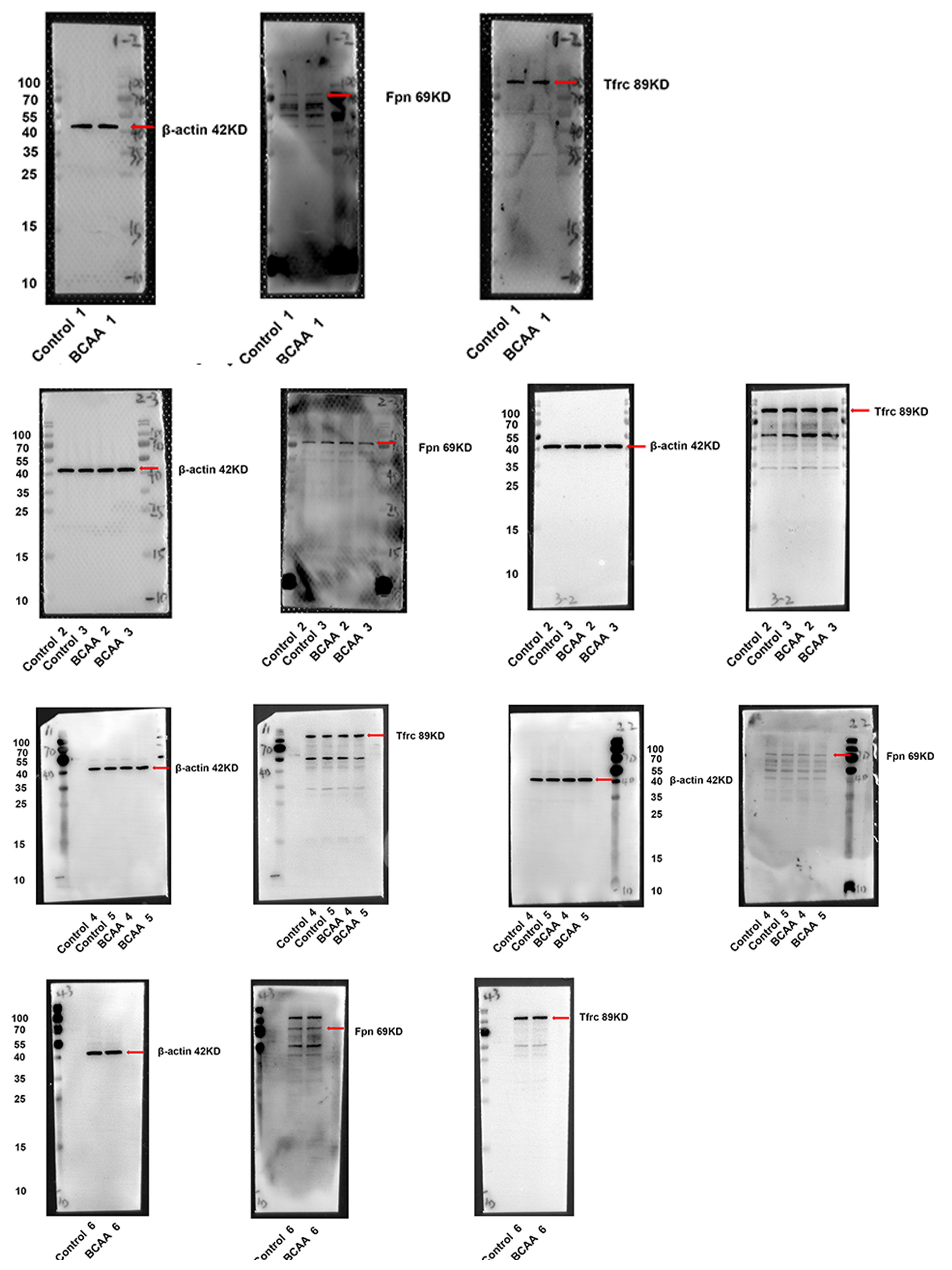


Figure. 9 The images of original, full-length Western blot gel of Supplementary Fig. 2 (f, g). Red asterisk indicates the objective band.


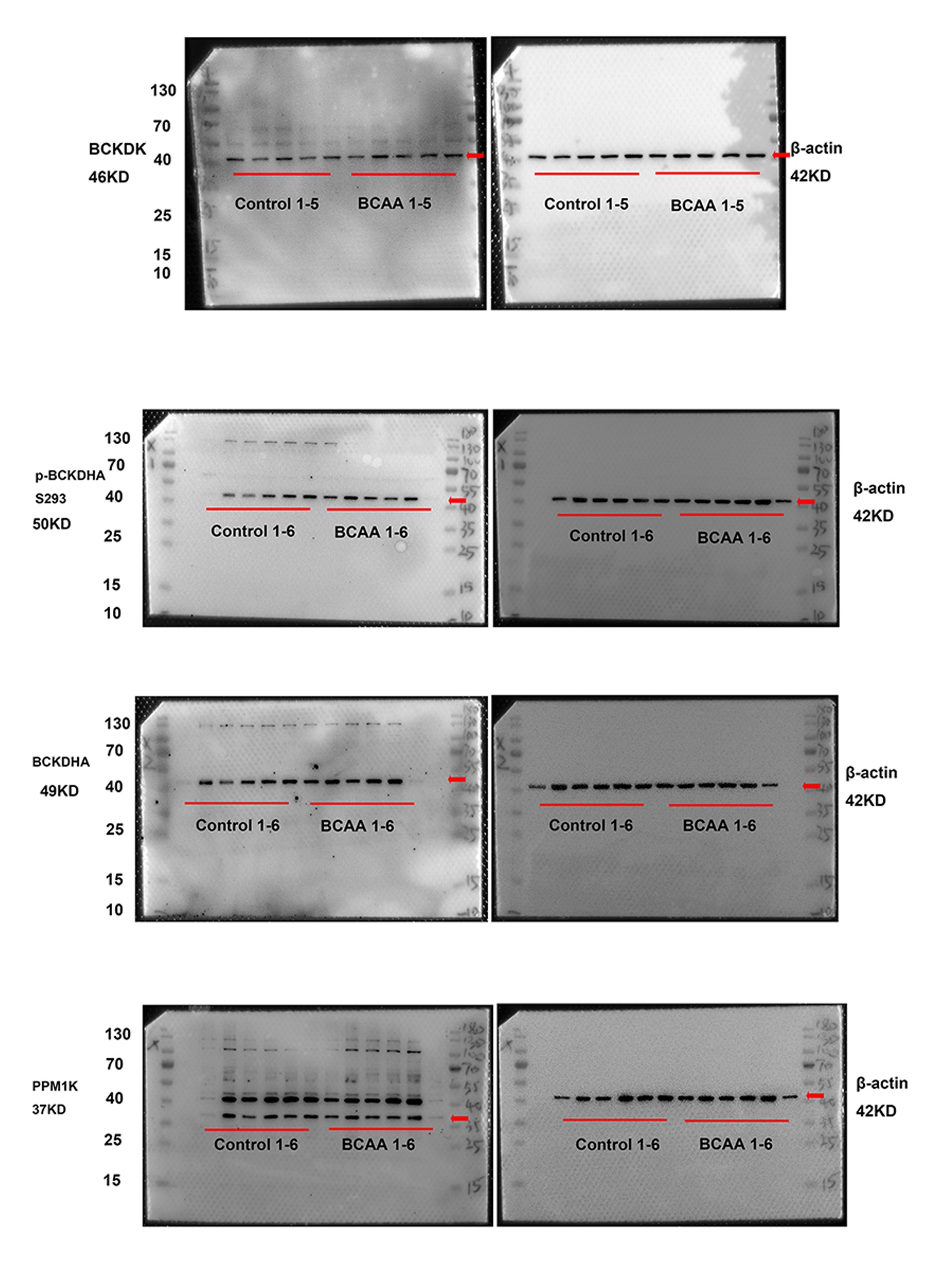


Figure. 10 The images of original, full-length Western blot gel of Supplementary Fig. 2 (h, i). Red asterisk indicates the objective band.
